# Novel Anthracycline Utorubicin for Cancer Therapy

**DOI:** 10.1002/ange.202016421

**Published:** 2021-06-01

**Authors:** Lorena Simón‐Gracia, Valeria Sidorenko, Ain Uustare, Ivan Ogibalov, Andrus Tasa, Olga Tshubrik, Tambet Teesalu

**Affiliations:** ^1^ Institute of Biomedicine and Translational Medicine University of Tartu Ravila 14b 50411 Tartu Estonia; ^2^ Toxinvent LLC Tiigi 61b 50410 Tartu Estonia; ^3^ Cancer Research Center Sanford-Burnham-Prebys Medical Discovery Institute 10901 North Torrey Pines Road La Jolla CA 92037 USA; ^4^ Center for Nanomedicine and Department of Cell Molecular and Developmental Biology University of California Santa Barbara Santa Barbara CA 93106 USA

**Keywords:** anthracycline, antitumor agents, polymersomes, targeted drug delivery, utorubicin

## Abstract

Novel anticancer compounds and their precision delivery systems are actively developed to create potent and well‐tolerated anticancer therapeutics. Here, we report the synthesis of a novel anthracycline, Utorubicin (UTO), and its preclinical development as an anticancer payload for nanocarriers. Free UTO was significantly more toxic to cultured tumor cell lines than the clinically used anthracycline, doxorubicin. Nanoformulated UTO, encapsulated in polymeric nanovesicles (polymersomes, PS), reduced the viability of cultured malignant cells and this effect was potentiated by functionalization with a tumor‐penetrating peptide (TPP). Systemic peptide‐guided PS showed preferential accumulation in triple‐negative breast tumor xenografts implanted in mice. At the same systemic UTO dose, the highest UTO accumulation in tumor tissue was seen for the TPP‐targeted PS, followed by nontargeted PS, and free doxorubicin. Our study suggests potential applications for UTO in the treatment of malignant diseases and encourages further preclinical and clinical studies on UTO as a nanocarrier payload for precision cancer therapy.

## Graphical abstract

Utorubicin (UTO) is a novel anthracycline with higher anticancer activity than doxorubicin in cultured cancer cells. Nanoencapsulation of UTO in polymeric vesicles functionalized with tumor‐penetrating peptides increases the cytotoxicity of the drug in receptor‐positive cells in vitro and, after systemic administration into mice bearing triple‐negative tumors, potentiates UTO accumulation in malignant tissue.

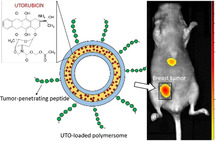

## Introduction

Anthracyclines, such as doxorubicin (DOX) and daunorubicin, have been among the most widely used cancer therapeutics for more than 30 years.^[1]^ Mechanistically, the four‐ring anthraquinone backbone of anthracyclines intercalates DNA and the sugar moiety bound through a glycosidic linkage interacts with base pairs in the minor groove.^[2]^ These drug interactions interfere with DNA synthesis, repair, and transcription, resulting in inhibition of cell replication and, eventually, cell death.[^3^, ^4^] Complexation of anthracyclines with DNA also inhibits the activity of topoisomerase II, an enzyme that manages DNA tangles and supercoils, impairing DNA repair.^[5]^ In addition, the quinone moiety in anthracyclines induces the formation of reactive oxygen species.[^6^, ^7^] Anthracyclines are used to treat many types of cancers, including leukemia, lymphomas, breast, gastric, uterine, ovarian, bladder, and lung cancers. Recognition that therapeutic application of anthracyclines is limited by side effects, especially by dose‐limiting cardiotoxicity,^[1]^ has led to extensive efforts to develop anthracycline derivatives and their nanoformulations of improved safety profile and broader therapeutic index. For example, synthetic 9‐aminoanthracycline amrubicin, which was designed for reduced cardiotoxicity,[^8^, ^9^] has higher antitumor activity than DOX.^[10]^ Anthracyclines have been covalently coupled to nanocarriers (e.g. polymeric, gold, silver, silica, and graphene oxide nanoparticles) using linkers sensitive to physiological stimuli such as modulation of surrounding pH and redox potential, or enzymatic processing.^[5]^ Unmodified anthracyclines have been encapsulated in polymeric, lipidic and inorganic nanoparticles and in dendrimers.^[11]^ DOX nanoformulated in PEGylated‐liposomes (Caelyx^TM^), developed in the late 1980s, is clinically approved for the treatment of multiple myeloma, Kaposi sarcoma, and breast and ovarian carcinoma.[^1^, ^12^] However, compared to the parental free drug, the anticancer efficacy of liposomal doxorubicin is not significantly higher[^13^, ^14^] and, despite improved safety profile, it still elicits side effects such as dermal toxicities and mucositis.^[15]^

The tumor accumulation of systemic drug nanocarriers is driven by structural and functional abnormalities in tumor‐feeding blood and lymphatic vessels that manifest in a phenomenon known as the enhanced permeability and retention (EPR) effect.^[16]^ However, the EPR effect is affected by the tumor location, type, and stage, and it shows wide inter‐ and intratumoral heterogeneity.^[17]^ Affinity targeting with ligands such as peptides and antibodies may allow for more efficient and uniform delivery of payloads to the tumor vessels and parenchyma than is possible by solely relying on EPR effect. In particular, tumor‐penetrating peptides (TPPs) can be used for precision parenchymal delivery of drugs in solid tumors.[^18^, ^19^, ^20^, ^21^] The LinTT1 (AKRGARSTA) is a TPP first recruited to p32 overexpressed on the surface of vascular and malignant cells and on tumor‐associated macrophages in many solid tumors,[^22^, ^23^, ^24^] followed by proteolytic processing to expose the C‐end Rule (CendR) motif of the peptide (AKRGAR, CendR motif underlined) to enable neuropilin‐1 (NRP‐1) binding, which activates a tumor penetration pathway.^[25]^ LinTT1 has been used for targeting therapeutic nanoparticles in the preclinical treatment of breast cancer,^[26]^ peritoneal carcinomatosis,^[27]^ and glioblastoma^[28]^ as well as for delivery of positron emission tomography (PET)‐active nanoparticles for early detection of triple‐negative breast tumors in mice.^[29]^

Here, in a quest to develop improved anthracyclines suitable for nanocarrier‐based delivery, we developed a novel 9‐aminoanthracycline prodrug, UTO. We report studies on its synthesis, comparative toxicity profiling on cultured malignant cells, as well as comparison of uptake and activity of free versus nanoformulated UTO.

## Results

### Synthesis of UTO

9‐Aminoanthracyclines have shown less cardiotoxicity and more controllable toxic effects than other anthracyclines such as DOX, daunorubicin and idarubicin.[^8^, ^9^] Anthracyclines undergo enzymatic reduction of the C‐13 that is catalyzed by a cytoplasmic carbonyl reductase and converts a carbonyl to a hydroxyl group.^[30]^ In the case of the 9‐aminoanthracycline amrubicin, the corresponding metabolite amrubicinol is up to 50 times more potent than the parent drug.^[31]^ The cytotoxic effect can be further increased by attaching a methylene group to the amino and hydroxyl groups (between respective nitrogen and oxygen atoms) of the 1,2‐aminoalcohol moiety of the daunosamine.^[32]^ The resulting oxazolidine cycle forms an anthracycline–DNA adduct that inhibits cell replication.^[33]^

**Scheme 1:**
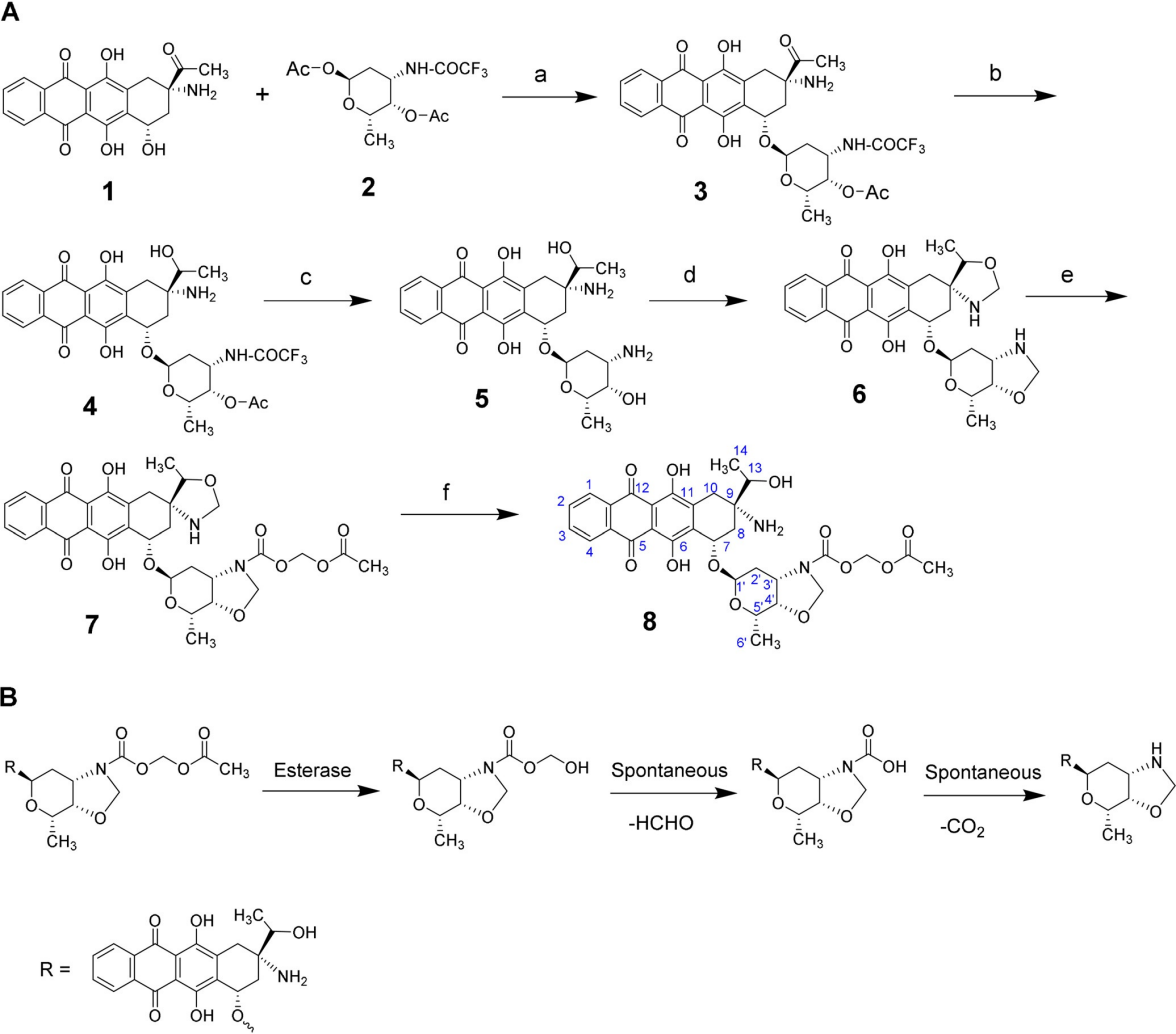
Synthesis and activation of UTO. A) Synthesis of utorubicin (compound **8**). a) Trimethylsilyl trifluoromethanesulfonate, 4 Å molecular sieves, 90 % yield; b) NaBH(OAc)_3_ in EtOH, 26 % yield; c) LiOH in THF/MeOH/H_2_O, 56 % yield; d) paraformaldehyde in CHCl_3_, 48 % yield; e) (acetyloxy)methylcarbonic acid, 4‐nitrophenyl ester; f) AcOH in MeCN/H_2_O, 18 % over 2 steps. B) Scheme of the deprotection of the UTO prodrug. Esterases hydrolyze the acetyl group from the acetyloxymethyl carbamate protecting group of UTO, followed by spontaneous decomposition of the hemiacetal. The oxazolidine cycle is then able to form the anthracycline–DNA adduct.

Here we developed a novel 9‐aminoanthracycline prodrug, UTO, that contains a hydroxyl group at the C‐13 position and an oxazolidine cycle in C‐3′, C‐4′ of the daunosamine moiety (Scheme 1 A). The active drug (compound **6**) was obtained following a four‐step procedure and its oxazolidine cycle was subsequently protected to form the prodrug UTO (compound **8**). The synthesis started from commercially available amrubicinone (aglycone part of amrubicin, **1**). Amrubicinone was glycosylated with protected aminosugar L‐daunosamine (1,4‐di‐*O*‐acetyl‐*N*‐trifluoroacetyl‐β‐L‐daunosamine, compound **2**) in the presence of trimethylsilyl trifluoromethanesulfonate to obtain compound **3**. The carbonyl group of compound **3** was reduced with sodium triacetoxyborohydride to yield compound **4**. In this step, other reduction conditions were tested (NaBH_4_, NaBH_3_CN) with unsuccessful results. The low yield of compound **4** could be partially due to compound **3** being not fully pure. Compound **4** was deprotected by cleavage of the N‐trifluoroacetyl and O‐acetyl groups from the L‐daunosamine moiety to obtain compound **5**. The oxazolidine cycle was then formed by reacting compound **5** with paraformaldehyde. The unreacted compound **5** was separated by filtration and the solution was concentrated and triturated with diethyl ether to obtain compound **6** (yield ≈48 %) that was used without further purification to prepare the prodrug. Compound 6 was characterized by ^1^H‐NMR, ^13^C‐NMR, and ^13^C‐DEPT studies (Figure S7–S10).

The oxazolidine cycle of compound **6** is unstable in aqueous media and undergoes fast hydrolysis under acidic conditions. Therefore, it was stabilized with a biocleavable protecting group^[34]^ by the formation of a carbamate bond through the nitrogen atom in the oxazolidine cycle. The acetyl group from the acetyloxymethyl carbamate protecting group is susceptible to esterases, followed by spontaneous decomposition of the resulting hemiacetal, resulting in exposure of reactive oxazolidine cycle (Scheme 1 B), which is able to form an anthracycline–DNA adduct.^[33]^ Carboxylesterases (CE) are widely distributed in the body and also found in tumors.^[35]^ The ability of CE to hydrolyze ester, amide, and carbamate groups to alcohol and carboxylic acid has been extensively described[^36^, ^37^] and is used for the activation of various antiviral, anticancer, and antibiotic prodrugs. For example, pentyl PABC‐Doxaz prodrug is hydrolyzed by carboxylesterase 2 (CES2) to the active formaldehyde‐Dox conjugate.^[38]^ The cleavage occurs via serine hydrolase mechanism followed by three spontaneous steps, ending in a second decarboxylation that, similarly to UTO hydrolysis, results in the free oxazolidine cycle.

The analog of amrubicin containing oxazolidine cycle (compound **6**) was reacted with 4‐nitrophenyl(acetyloxy)methylcarbonate (Scheme 1 A) to obtain compound **7**. Under these conditions, only the oxazolidine cycle in the sugar moiety is available for reaction as the nitrogen atom at C9 is highly sterically hindered. Compound **7** was not isolated and the reaction was mixed with acetic acid, and stirred to hydrolyze the unreacted oxazolidine cycle. The final purified prodrug (UTO, compound **8**) was obtained with an 18 % yield (with respect to compound **6**) at >96 % purity (Figure S15) and was characterized by NMR (Figure S11–14) and HMRS (Figure S16). The low yield of **8** might be due to partial hydrolysis of the oxazolidine in the sugar before reaction with 4‐nitrophenyl‐(acetyloxy)‐methylcarbonate, yielding the analogue of compound **8** without oxazolidine cycle. The optimization of the synthesis steps that resulted in low yields (from compound **3** to **4** and from **6** to **8**) will be a subject of follow‐up studies.

### Cytotoxicity of UTO on Cultured Cancer Cell Lines

**Figure 1:**
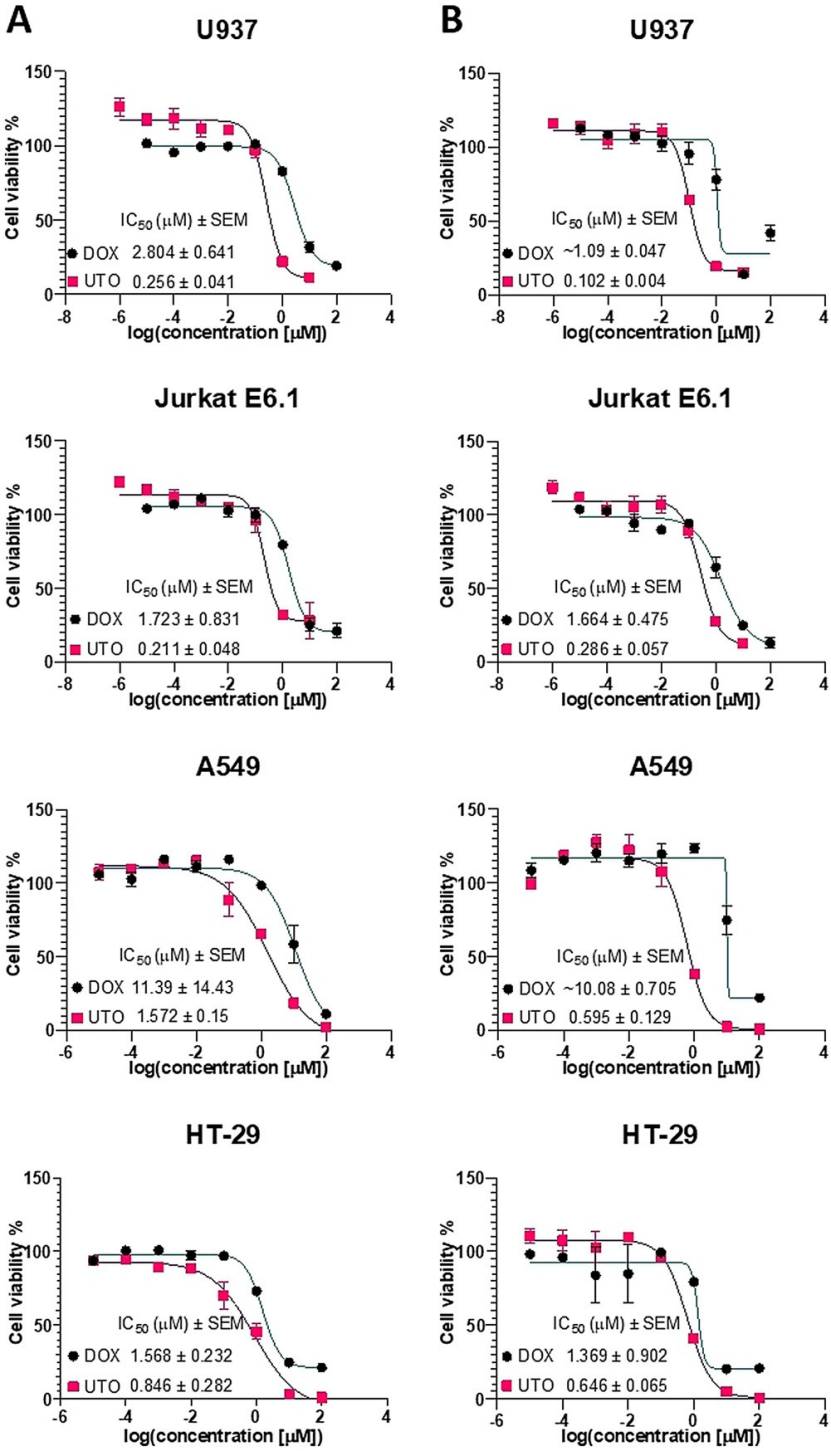
Cytotoxicity of UTO on cultured cancer cell lines using MTS assay. Viability of the cancer cells incubated with indicated concentrations of UTO and DOX for 30 (A) or 90 (B) min and chase in drug‐free medium. IC_50_ (half‐maximal inhibitory concentration) was determined using log(inhibitor) versus response–variable slope (four parameters) model (GraphPad Prism version 5.03 for Windows, GraphPad Software, La Jolla California USA). The standard error of the mean (SEM) of the IC_50_ is shown. The cells were incubated with the anthracyclines, washed, and cultured for 24 h (U937 and Jurkat E6.1 cells in suspension) or 72 h (adherent A549 and HT‐29 cells) followed by the viability assay. As controls, 1 % water, or 1 % DMSO (solvents used to dissolve DOX and UTO respectively) v/v in cell culture medium were used. MTS assay results were plotted as a curve of the percentage of viable cells (taking the viability of the nontreated control cells as 100 %) versus log concentration of tested sample. Error bars represent the mean standard deviation. *N*=3.

We first performed comparative studies on the effect of treatment with UTO and DOX on the viability of a panel of cultured cell lines: U937 monoblast‐like human histiocytic lymphoma cells, Jurkat E6.1 human T‐lymphocyte from acute T‐cell leukemia cells, A549 human lung carcinoma cells, and HT‐29 human colorectal adenocarcinoma cells. The cytotoxicity was tested after 30 and 90 min of incubation of the cells with the drugs and follow‐up culture for 24 h (U937 and Jurkat E6.1 cells in suspension), or 72 h (adherent A549 and HT‐29 cells). In all tested cell lines, UTO had a more potent effect on reducing cell viability than DOX (Figure 1). The highest difference in the IC_50_ between the UTO and DOX was observed for A549 (with UTO ≈16‐fold more effective) and the lowest for HT‐29 cells (with UTO ≈2‐fold more effective).

### Generation of Peptide‐Guided UTO‐Polymeric Nanocarriers

**Figure 2:**
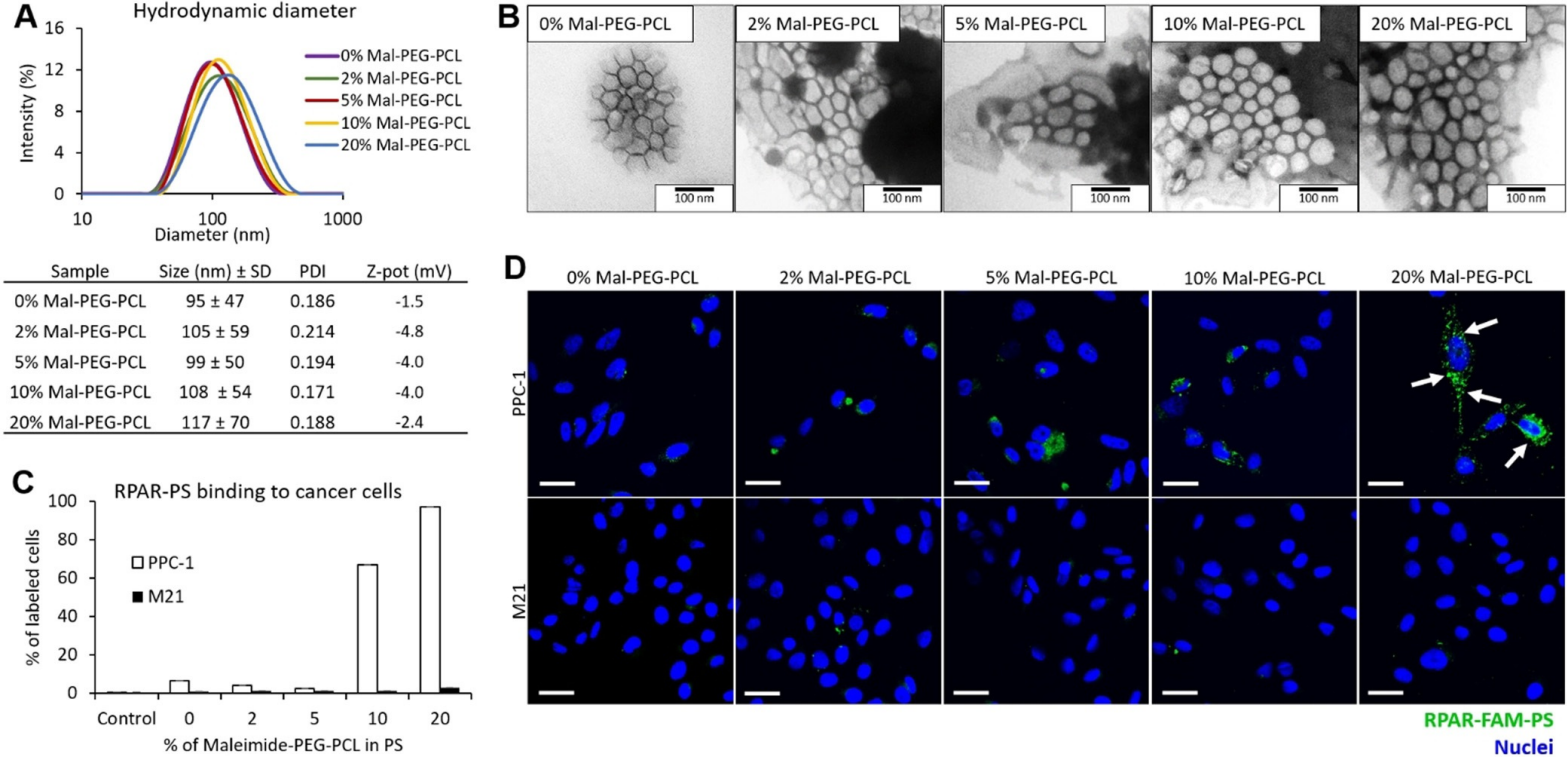
Optimization of PS for peptide‐guided delivery. A) Characterization of PS over a range of maleimide‐PEG‐PCL (Mal‐PEG‐PCL) (0; 2; 5; 10 and 20 %): DLS graphs and characteristics of size, PDI, and Z‐potential of the PS. B) TEM of the FAM‐PS. Scale bar=100 nm. C) Binding of RPAR‐FAM‐PS to cultured PPC‐1 and M21 cells. Attached cells were incubated with PS samples for 1 h, washed, detached, and flow cytometry was used to quantify the binding of RPAR‐FAM‐PS to PPC‐1 (NRP‐1^+^) and M21 (NRP1^−^) cells. PS were prepared incorporating the indicated percentage of Mal‐PEG‐PCL. The graph represents the percentage of labeled cells. Control is the cells without incubation with PS. *N*=3. Error bars indicate ± standard error of the mean (± SEM). D) Fluorescence confocal microscopy imaging of attached PPC‐1 and M21 cells incubated with RPAR‐FAM‐PS samples for 1 h. The PS were labeled with FAM (green signal) and the nuclei were stained with DAPI (blue signal). Scale bar=50 μm.

Recognition that hydrophobicity of UTO may limit its therapeutic application as a free drug, prompted us to evaluate it as an anticancer payload of peptide‐guided polyethylene glycol‐polycaprolactone (PEG‐PCL) polymersomes (PS). We first optimized the density of the prototypic CendR peptide RPARPAR (RPAR) on the surface of PS for targeting of NRP‐1^+^ cells. The density of RPAR on the PS surface was modulated by varying the content of maleimide‐PEG‐PCL relative to the whole amount of copolymer between 0 and 20 %. As the peptide conjugation occurs through the formation of a thioether bond between the thiol group of the cysteine added to the N‐terminus of the RPAR peptide (Cys‐RPAR) and the maleimide group of the copolymer, the number of surface maleimide groups determines the peptide density on PS nanoparticles. PS were prepared by film hydration method using a protocol adapted from a previous study.^[29]^ PS contained 5 % of FAM‐labeled‐PEG‐PCL (FAM‐PEG‐PCL) for fluorescence‐based tracing. The hydrodynamic diameter of the PS measured by dynamic light scattering (DLS) (105 nm) and the polydispersity index (0.19) were similar for all vesicle preparations independent of peptide coating and density (Figure 2 A). Transmission electron microscopy (TEM) showed that FAM‐labeled RPAR‐PS (RPAR‐FAM‐PS) appear as homogeneous spherical vesicles (Figure 2 B). We next studied the release of UTO from polymersomes in PBS during incubation at 37 °C. After 48 h, less than 2 % of UTO was released from the PS (Figure S19).

To study the effect of peptide coating on cellular uptake of PS, we incubated FAM‐labeled PS with cultured PPC‐1 and M21 cells—a well‐established system that has been used for CendR peptide based affinity targeting in vitro studies.[^40^, ^41^] PPC‐1 human primary prostate cancer cells have elevated expression of NRP‐1 (Figure S20). In contrast, M21 human melanoma cells are negative for NRP‐1 expression (Figure S20).[^39^, ^40^] PPC‐1 and M21 cells were incubated with RPAR‐FAM‐PS for 1 h, washed, detached, and analyzed by flow cytometry for the FAM positivity. In PPC‐1 cells, an increase in surface density of RPAR peptide resulted in a progressive increase in binding of RPAR‐FAM‐PS (Figure 2 C). RPAR‐PS formed using 20 % maleimide‐PEG‐PCL showed the highest uptake, with nearly all cells positive for FAM fluorescence. In contrast, the binding of RPAR‐FAM‐PS to the M21 cells was low and not affected by the surface density of the RPAR peptide. The zeta potential of all PS samples was neutral (Figure 2 A), suggesting that the differential binding of PS was not due to differences in PS surface charge. In line with flow cytometry studies, confocal microscopy demonstrated the presence of the green signal representing RPAR‐FAM‐PS only in PPC‐1 and not in M21 cells (Figure 2 D). The highest RPAR‐FAM‐PS signal was associated with PPC‐1 cells incubated with PS assembled using 20 % maleimide‐PEG‐PCL. Already at 1 h time point, the RPAR‐FAM‐PS was predominantly seen in vesicular structures in the cytoplasm, indicating cellular uptake (Figure 2 D, arrows).

**Figure 3:**
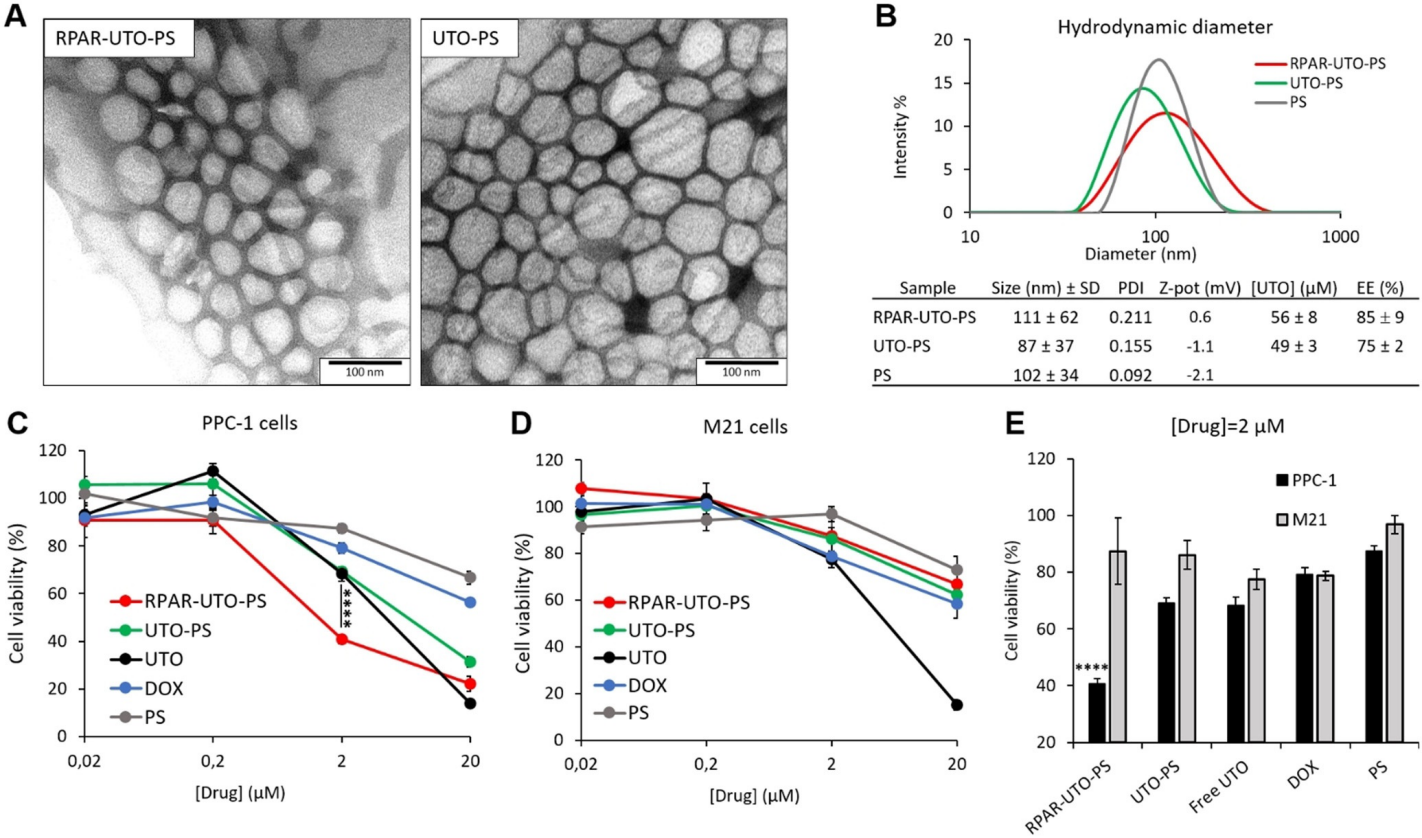
Effect of free and nano‐encapsulated UTO on the viability of cultured cells with different NRP‐1 expression status. A) TEM imaging of RPAR‐UTO‐PS and UTO‐PS. Scale bar=100 nm. B) Size distribution, UTO concentration, and encapsulation efficiency of PS samples. C, D) Percentage of viable PPC‐1 (C) and M21 cells (D) after 30 min incubation with PS formulations at 0.02, 0.2, 2, and 20 μM of UTO and 48 h follow‐up incubation. E) Percentage of viable PPC‐1 and M21 cells after 30 min treatment with PS formulations at 2 μM of UTO and 48 h follow‐up incubation. P values for the viable PPC‐1 and M21 cells after 30 min treatment with PS formulations at 2 μM of UTO or DOX are shown in Table 1 in the Supporting Information. Error bars indicate ± SEM; **** *p*<0.0001, *N*=5.

For loading UTO in PS, we used the film hydration method, followed by size exclusion chromatography to remove the non‐encapsulated drug. The morphology (observed by TEM, Figure 3 A) and hydrodynamic diameter of UTO‐loaded RPAR‐functionalized PS (RPAR‐UTO‐PS), UTO‐loaded non‐targeted PS (UTO‐PS), and non‐loaded “empty” PS (PS) were similar (Figure 3 B). The zeta potential of the PS was neutral and the UTO concentration in UTO‐PS samples was ≈50 μM with an encapsulation efficiency (EE) of ≈80 % (Figure 3 B)—dramatically higher than 1 % observed for hydrophilic doxorubicin⋅HCl (DOX) (Figure S21).

### Cytotoxicity of UTO‐Loaded RPAR‐Targeted PS on Cultured Cancer Cells

We next performed a comparative evaluation of the effect of free UTO and DOX, as well as RPAR‐guided and control UTO‐PS on the viability of PPC‐1 and M21 cells. The cells were treated for 30 min with the compounds, followed by 48 h follow‐up incubation in fresh culture medium and MTT viability assay. In both cell lines, free UTO was more toxic than free DOX. (Figure 3 C and D). For nano‐encapsulated UTO, the cytotoxic effect on cultured cells was dependent on targeting peptide functionalization and on the expression of peptide receptor, NRP‐1. RPAR‐guided UTO‐PS (at 2 μM UTO) showed significant potentiation of antiproliferative effect compared to nontargeted UTO‐PS at the same drug loading (≈40 % vs. ≈70 % cell viability; Figure 3 C and E).

### In Vivo Homing of TPP‐PS

**Figure 4:**
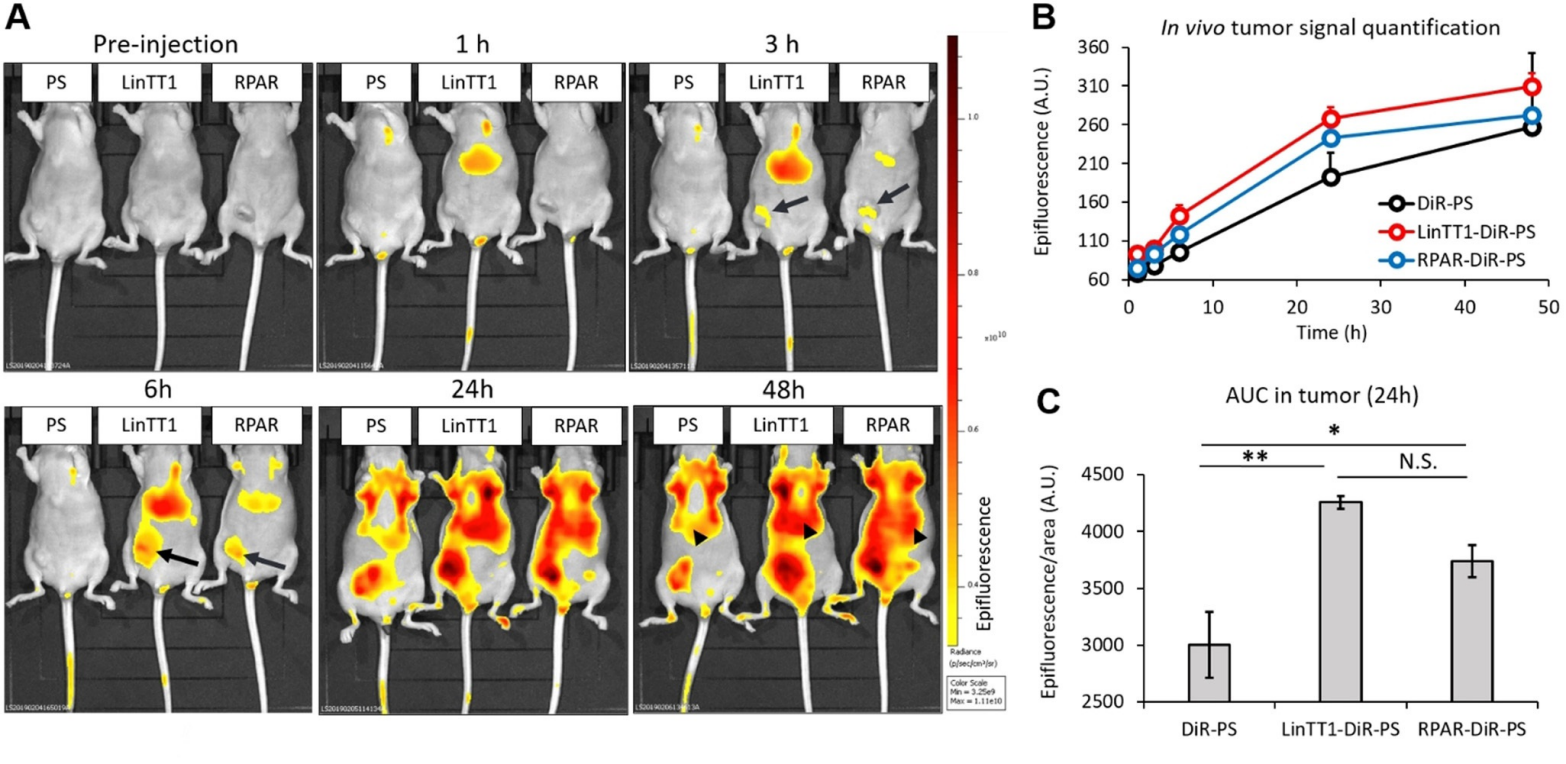
Systemic peptide‐targeted PS home to MCF10CA1a breast tumors. A) Live imaging of MCF10CA1a tumor‐bearing mice injected with LinTT1‐DiR‐PS (LinTT1), RPAR‐DiR‐PS (RPAR), or non‐targeted DiR‐PS (PS) at indicated post‐administration time points. The signal of DiR‐PS in the tumor is pointed out with arrows and in the liver with arrowheads. B) Quantitation of accumulation of DiR‐labeled PS in the tumor tissue at different post‐injection time points. The fluorescence signal was quantified from the IVIS images. For 1, 3, 6, and 24 h time points *N*=3; for 48 h RPAR‐DiR‐PS *N*=1, for LinTT1‐DiR‐PS and DiR‐PS *N*=2. Error bars indicate +SEM. C) The area under the curve (AUC) in the tumors of LinTT1‐DiR‐PS, RPAR‐DiR‐PS, and DiR‐PS at 24 h post‐injection. *N*=3, error bars=± SEM, ** *p*<0.01, * *p*<0.05, N.S.=not significant.

**Figure 5:**
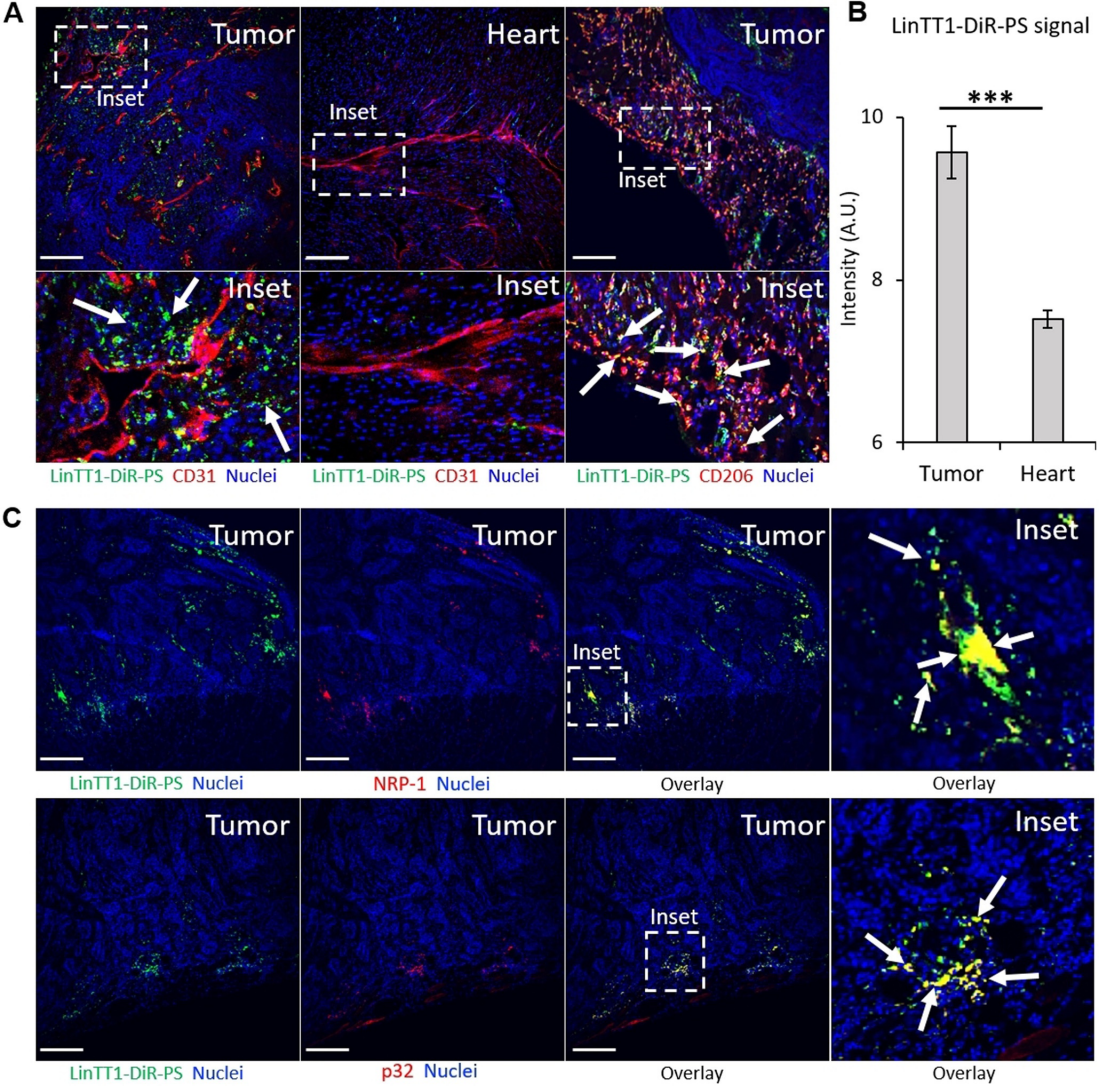
Tissue distribution of LinTT1‐DiR‐PS and UTO‐PS in MCF10CA1a tumor‐bearing mice. A) Confocal fluorescence imaging of tumor and heart of TNBC bearing mice injected with LinTT1‐DiR‐PS. Tissues were extracted at 48 h post‐injection of PS, sectioned, and immunostained for FAM, CD31, and CD206; stained with DAPI. Green signal represents LinTT1‐DiR‐PS (FAM), red signal represents blood vessels (CD31) or mannose receptor CD206, and blue signal represents nuclei (DAPI); scale bar=200 μm. B) LinTT1‐DiR‐PS signal quantification in heart and tumor at 48 h post‐injection, *N*=6 (different areas of the same section), *** *p*<0.001. Error bars indicate ± standard error of the mean (± SEM). C) Colocalization of LinTT1‐DiR‐PS with NRP‐1 or p32 proteins in breast tumor tissues.

We next studied in vivo biodistribution of systemic LinTT1‐, RPAR‐, and nontargeted PS loaded with near‐infrared DiR dye (LinTT1‐DiR‐PS, RPAR‐DiR‐PS, and DiR‐PS) as a model imaging payload that mimics UTO in being hydrophobic and could be used for initial calibration before using the actual drug. The DiR‐loaded PS appeared spherical (Figure S22) with an average hydrodynamic diameter similar to the previous PS formulations (average size: 116 nm, PDI ≈0.15), suggesting that the presence of dye in the PS membrane does not affect the structure of the nanovesicles. For tumor induction, the mice were orthotopically injected with human MCF10CA1a triple‐negative breast cancer (TNBC) cells. MCF10CA1a cells and activated stromal cells in MCF10CA1a tumor lesions overexpress surface p32,^[42]^ thus rendering LinTT1 (a ligand for p32) a suitable peptide to target this tumor. Moreover, functionalization of therapeutic nanoparticles with LinTT1 peptide was found to improve their anticancer efficacy in experimental therapy of MCF10CA1a breast tumor.^[26]^ LinTT1‐DiR‐PS, RPAR‐DiR‐PS, and DiR‐PS were injected intravenously (i.v.) into TNBC mice and live fluorescence imaging was performed at 1, 3, 6, 24, and 48 h post‐injection. Targeting with LinTT1 and RPAR peptides increased tumor homing of DiR‐PS (Figure 4). LinTT1‐ and RPAR‐DiR‐PS were detected in tumors already at 3 h after administration (arrows in Figure 4 A), whereas nontargeted DiR‐PS became detectable after 24 h post‐injection. Compared to nontargeted DiR‐PS, the area under the curve (AUC) in tumor lesions at 24 h for peptide‐targeted DiR‐PS was elevated by ≈42 % (for LinTT1‐DiR‐PS) and ≈25 % (for RPAR‐DiR‐PS) (Figure 4 C). As expected, the LinTT1‐, RPAR‐, and non‐targeted DiR‐PS were also observed in the organs of the reticuloendothelial system: the liver (arrowheads in Figure 4 A) and the spleen. Forty‐eight hours after PS injection, tumors and control tissues were excised, sectioned, and the microscopic localization of PS was analyzed. We observed the accumulation of LinTT1‐DiR‐PS in the extravascular tumor parenchyma (Figure 5 A) and their co‐localization with the known receptors of LinTT1 peptide, p32, and NRP‐1 (Figure 5 C). We studied the co‐localization of LinTT1‐DiR‐PS with the CD206 receptor expressed on M2 protumoral macrophages known to promote growth and metastasis of malignant cells in solid tumors.^[43]^ We observed that LinTT1‐DiR‐PS accumulate in M2 macrophages in the tumor (Figure 5 A). Cardiotoxicity is the dose‐limiting toxicity of anthracyclines and we studied the accumulation of LinTT1‐DiR‐PS in the heart of TNBC‐bearing mice. As shown in Figure 5 A and B, the myocardial tissue contained a significantly lower signal of the LinTT1‐DiR‐PS than the malignant tissue.

### Tumor Accumulation of UTO Loaded in PS

Having established conditions for enhanced tumor accumulation of LinTT1‐targeted PS, we next studied the biodistribution of UTO loaded in PS. LinTT1‐targeted and nontargeted PS loaded with UTO (LinTT1‐UTO‐PS, UTO‐PS), and free DOX were i.v. injected into mice bearing orthotopic MCF10CA1a tumors. DOX served as a surrogate for free UTO, which could not be used because of its poor water solubility.

**Figure 6:**
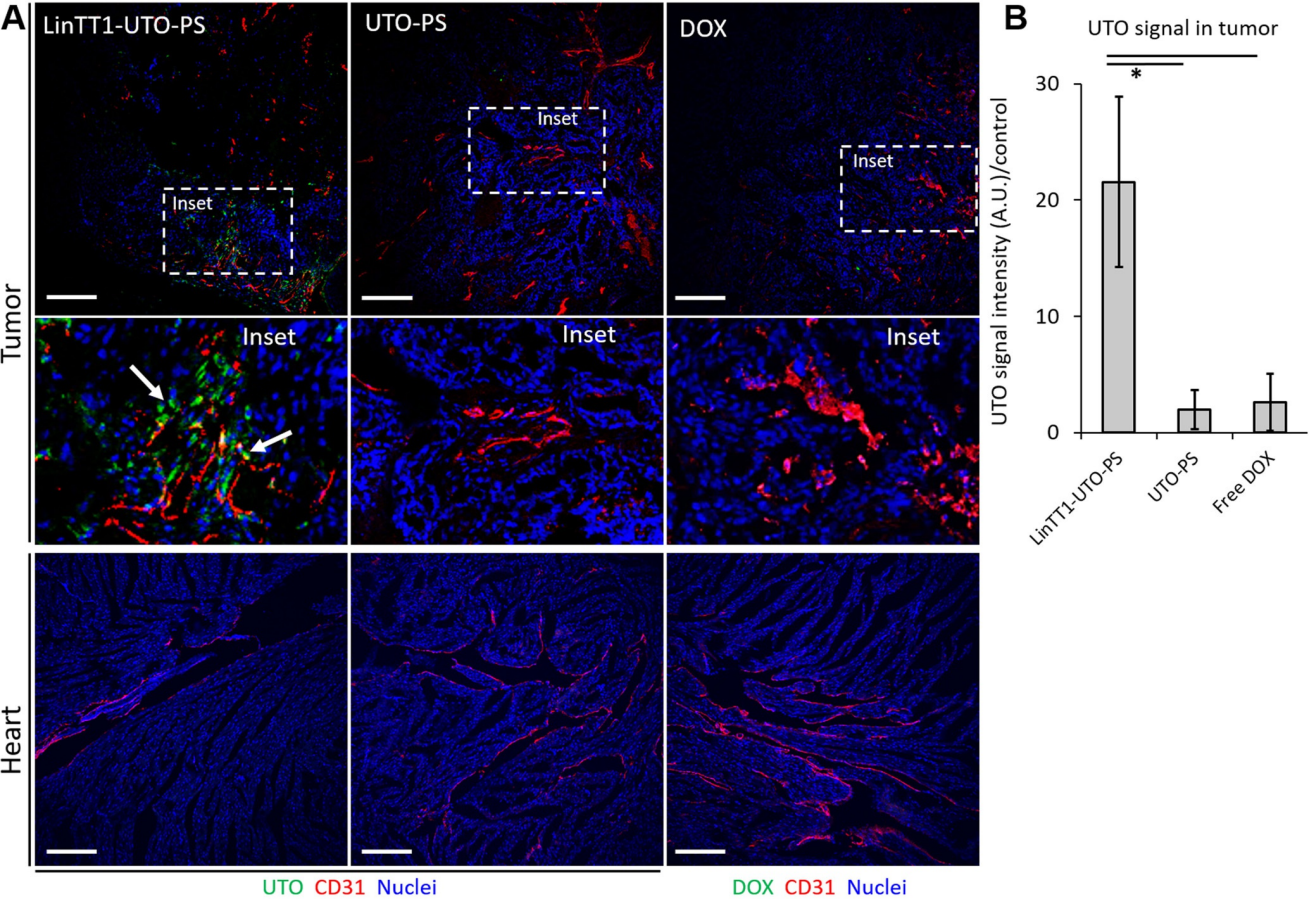
A) Confocal fluorescence imaging of MCF10CA1a tumor and heart sections of mice injected with LinTT1‐UTO‐PS, UTO‐PS, and free DOX. Samples were collected at 24 h post‐injection, sectioned, immunostained for CD31, and stained with DAPI. Green: UTO and DOX; red: blood vessels (CD31); blue: nuclei (DAPI). Scale bar=200 μm. B) LinTT1‐UTO‐PS signal quantification in tumors at 24 h post‐injection. * *p*<0.05. Error bars indicate ± SEM, *N*=3.

As shown in Figure 6 A and B, there was a significantly higher tumor accumulation of UTO in mice injected with LinTT1‐UTO‐PS in comparison with other samples. UTO signal co‐localized with CD31‐positive blood vessels; in addition, some signal was seen in the perivascular space with LinTT1‐UTO‐PS, but not with UTO‐PS, suggesting that the drug loaded in the LinTT1‐PS had extravasated and spread into the tumor tissue (Figure 6 A, inset). Importantly, no UTO signal was observed in the heart of the LinTT1‐UTO‐PS treated mice (Figure 6 A). These observations suggest that the encapsulation of UTO in LinTT1‐PS enhances the specific tumor accumulation of the drug.

## Discussion

Anthracycline drugs are among the most widely used antitumor drugs. However, their side effects, such as cardiotoxicity, bone marrow depression, and gastrointestinal disturbances, limit their therapeutic application at doses sufficient to eradicate malignant lesions. Here we report the synthesis of novel anthracycline, UTO, and show that it is more effective in killing cultured cancer cells than the widely used DOX. Furthermore, we show that UTO, which is too hydrophobic to be used in vivo as such, can be loaded with high efficiency in polymersome nanocarriers (PS), and that UTO‐PS functionalized with homing peptides accumulate selectively in cultured tumor cells expressing the receptor for the peptide and, upon systemic administration, in tumor lesions in vivo.

UTO was designed to achieve improved anticancer efficacy with lower side effects and good tolerability. Similar to amrubicinol, the reduced metabolite of amrubicin (the first synthetic anthracycline clinically approved for the treatment of lung cancer), UTO contains an amino group in the 9‐position of the anthraquinone and a hydroxyl group in the C‐13 position. Whereas both UTO and DOX include a daunosamine moiety, in UTO it contains an oxazolidine cycle that can facilitate the formation of DNA adducts.[^32^, ^33^, ^44^] The acetyloxymethyl carbamate group of UTO is hydrolyzed by esterases to expose the reactive oxazolidine cycle. The four‐ring structure of UTO intercalates the DNA and the oxazolidine cycle binds via its methylene carbon of guanine residues to block DNA‐replication and transcription processes. Amrubicin and amrubicinol, which induce cell growth inhibition by stabilizing the topoisomerase II‐DNA complex, are less potent DNA intercalators than DOX.^[45]^

About 40 % of approved drugs and nearly 90 % of drug candidates are poorly soluble in aqueous solutions. Whereas hydrophobicity of free UTO poses a formulation challenge and is likely to limit its bioavailability, we hypothesized that its nanoformulation can bypass these problems. Since a pioneering 1984 study on nanosized polymeric self‐assemblies as hydrophobic drug solubilizers,^[46]^ nano‐delivery systems have been used to improve solubility of different hydrophobic drugs, such as paclitaxel. Abraxane®, a paclitaxel albumin‐bound NP formulation with a particle size of ≈130 nm, was approved by the FDA in 2005 for the treatment of metastatic breast cancer.^[47]^ In the current study, we showed that PS can be used to formulate hydrophobic UTO for systemic application without the use of toxic solvents such as Cremophor EL. Besides serving as a formulation aid for hydrophobic drugs, PS are well suited for their precision delivery. First, PEG‐PCL PS are fully biocompatible.^[48]^ Second, PS can be readily functionalized with affinity targeting ligands for the site‐ and cell type‐specific delivery. Third, although not made use of here, different physicochemical triggers, such as change in pH, can be used to actuate disassembly of internalized PS and cargo release.[^49^, ^50^, ^51^] Indeed, we observed improved homing of systemic TPP‐functionalized PS in the breast tumors. We observed a prominent homing of LinTT1‐PS in the M2‐differentiated tumor‐associated macrophages (M2‐TAMs), in particular at the tumor rim. The depletion of protumoral M2‐TAMs with DOX‐loaded nanoparticles has been reported to result in suppression of tumor growth.^[52]^ Moreover, drug‐loaded TAMs act as a drug reservoir that releases the drug over extended periods of time.^[53]^ Therefore, targeting M2‐TAMs with UTO‐loaded PS functionalized with LinTT1 (or other M2 TAM specific peptides[^54^, ^55^]) may become another anticancer therapeutic strategy.

Interestingly, after systemic administration of LinTT1‐UTO‐PS in breast tumor mice, no UTO signal was observed in the heart. In reported biodistribution studies using amrubicin, DOX, daunorubicin, and their reduced 13‐hydroxy metabolites, it was found that amrubicinol is more selective for tumors than the rest of anthracycline metabolites.^[56]^ Therefore, the lower cardiotoxicity of amrubicin might be a consequence of the lower heart accumulation of its active metabolite. The intrinsic tumor selectivity of 13‐hydroxy anthracyclines in combination with the precision nano‐delivery of the drug may result in a potent antitumor activity and low side effects. The therapeutic activity of the UTO‐loaded TPP‐PS will be a subject of follow‐up studies.

## Conclusion

We synthesized a new anthracycline, utorubicin (UTO), with higher cell‐killing activity than DOX on cultured tumor cell lines. To deal with the limitations posed by the hydrophobicity of UTO, we developed protocols for UTO encapsulation in peptide‐guided, biocompatible, and biodegradable polymersome nanoparticles. The nanoformulated UTO functionalized with tumor targeting peptides showed selective internalization and killing of cultured peptide‐receptor positive cells and accumulation in tumors upon systemic administration in vivo.

## Conflict of interest

T.T. is the inventor of patents on CendR peptides and shareholders of Cend Therapeutics Inc., a company that holds a license for the CendR peptides and is developing the peptides for clinical use. VS, LSG, OT, AT, AU, and TT are inventors of a patent application on Utorubicin (application TD 40725 / ST).

## Supporting information

As a service to our authors and readers, this journal provides supporting information supplied by the authors. Such materials are peer reviewed and may be re‐organized for online delivery, but are not copy‐edited or typeset. Technical support issues arising from supporting information (other than missing files) should be addressed to the authors.

Supplementary
