# Supplementary material for: Novel Anthracycline Utorubicin for Cancer Therapy
Source: Angew Chem Weinheim Bergstr Ger. 2021 Jun 1;133(31):17155–64. doi: 10.1002/ange.202016421 (PMC10947310; doi:10.1002/ange.202016421)
Supplement: Supplementary file 1 — Supplementary [file ANGE-133-17155-s001.pdf]

Angewandte Chemie
Eine Zeitschrift der Gesellschaft Deutscher Chemiker

Supporting Information

Novel Anthracycline Utorubicin for Cancer Therapy

Lorena Simón-Gracia[+], Valeria Sidorenko[+], Ain Uustare, Ivan Ogibalov, Andrus Tasa, Olga Tshubrik, and Tambet Teesalu**

ange_202016421_sm_miscellaneous_information.pdf

Author Contributions

L.S. Conceptualization: Equal; Data curation: Equal; Formal analysis: Equal; Funding acquisition: Supporting; Investigation: Lead; Methodology: Equal; Supervision: Lead; Validation: Lead; Writing—original draft: Equal; Writing—review & editing: Equal
V.S. Data curation: Equal; Formal analysis: Equal; Methodology: Equal; Writing—original draft: Equal
A.U. Conceptualization: Equal; Data curation: Equal; Formal analysis: Equal; Investigation: Equal; Methodology: Equal; Writing—review & editing: Supporting
I.O. Conceptualization: Supporting; Methodology: Supporting
A.T. Conceptualization: Supporting; Funding acquisition: Equal; Resources: Equal; Writing—review & editing: Supporting
O.T. Conceptualization: Supporting; Funding acquisition: Equal; Resources: Equal; Writing—review & editing: Supporting
T.T. Funding acquisition: Equal; Project administration: Lead; Resources: Equal; Supervision: Supporting; Writing—original draft: Supporting; Writing—review & editing: Supporting.

Supporting Information
Table of contents

EXPERIMENTAL PROCEDURES 3

MATERIALS 3

SYNTHESIS OF UTORUBICIN (UTO) 3

SYNTHESIS OF COMPOUND 3 3

SYNTHESIS OF COMPOUND 4 3

SYNTHESIS OF COMPOUND 5 4

SYNTHESIS OF COMPOUND 6 4

SYNTHESIS OF COMPOUND 8 4

SYNTHESIS OF FLUORESCEIN-PEG-PCL (FAM-PEG-PCL) 5

SYNTHESIS OF PEPTIDE-FUNCTIONALIZED POLYMERSOMES (PS) 5

NANOENCAPSULATION OF UTO 5

NANOENCAPSULATION OF DOXORUBICIN (DOX) 5

SYNTHESIS OF DIR-LABELLED PS 5

PURIFICATION OF PS 5

CHARACTERIZATION OF PS 6

EFFICIENCY OF THE PEPTIDE CONJUGATION TO PS 6

CELL CULTURE 6

CELLULAR BINDING ASSESSMENT BY FLOW CYTOMETRY 6

CELLULAR UPTAKE ASSESSMENT BY FLUORESCENCE CONFOCAL MICROSCOPY 7

ASSESSMENT OF IN VITRO CYTOTOXICITY 7

DRUG RELEASE STUDY 7

IN VIVO EXPERIMENTS 7

BIODISTRIBUTION OF PS FUNCTIONALIZED WITH TUMOR PENETRATING PEPTIDES 8

TISSUE IMMUNOFLUORESCENCE AND CONFOCAL MICROSCOPY 8

STATISTICAL ANALYSIS 8

SUPPLEMENTARY FIGURES 9

REFERENCES 34

AUTHOR CONTRIBUTIONS 34

Experimental Procedures

Materials

Amrubicinone (1, (7S, 9S)-9-acetyl-9-amino-7,8,9,10-tetrahydro-6,7,11-trihydroxy-5,12-naphthacenedione) was purchased from Abydos Scientific (China), 1,4-di-O-acetyl-N-trifluoroacetyl-β-L-daunosamine (2) was prepared as previously described[1] and characterized by 1H- and 13C-NMR (Figure S1, S2). The 4-nitrophenyl-(acetyloxy)-methylcarbonate was purchased from Accel Pharmatech (US), other reagents were purchased from TCI Europe (Belgium) and Sigma-Aldrich (Germany).
The copolymers polyethylene glycol-polycaprolactone (PEG_{5000}-PCL_{10000}; M_w 5,000 and 10,000 respectively; from now PEG-PCL) and maleimide-PEG_{5000}-PCL_{10000} (mal-PEG-PCL) were purchased from Advanced Polymer Materials Inc., Montreal, Canada.
5(6)-carboxyfluorescein-Cystein (FAM-Cys) and the peptides Ac-Cys-Ahx-RPARPAR-OH (Cys-RPAR) (Ahx is aminohexanoic acid linker), FAM-Cys-Ahx-RPARPAR-OH (FAM-Cys-RPAR), Ac-Cys-Ahx-AKRGARSTA-NH_2 (amidated at C-terminus, Cys-LinTT1) and FAM-Cys-Ahx-AKRGARSTA-NH_2 (amidated at C-terminus, FAM-Cys-LinTT1) were purchased from TAG Copenhagen, Denmark. XenoLight DiR (1,1'-dioctadecyltetramethyl indotricarbocyanine iodide) dye was purchased from PerkinElmer, USA. Amicon Ultra 0.5 mL centrifugal filters with MW cut-off of 100 kDa were purchased from Millipore, Sigma, US. Copper grids for transmission electron microscopy (TEM) (Formvar/Carbon, 300 Mesh Cu) were purchased from Agar Scientific, UK. Doxorubicin, isopropanol (2-propanol), dimethylformamide (DMF), 5-Methylphenazinium methyl sulfate (PMS), phosphotungstic acid, 3-(4,5-dimethylthiazol-2-yl)-2,5-diphenyltetrazolium bromide reagent (MTT), Tween-20, paraformaldehyde (PFA), Triton-X, size exclusion columns (inner diameter 1.0 cm, length 20 cm, bed volume 16 mL), and Sepharose (Sepharose 4B) were purchased from Sigma-Aldrich, Germany. Dulbecco's Modified Eagle Medium (DMEM) and phosphate-buffered saline (PBS) pH 7.4 were purchased from Lonza, Belgium. Trypan Blue stain was purchased from Gibco, Thermo Fischer Scientific, USA. Bovine serum albumin (BSA) and fetal bovine serum (FBS) were purchased from Capricorn Scientific, Germany. CellTiter 96®AQueaus MTS Reagent Powder was purchased from Promega, USA.

Synthesis of Utorubicin (UTO)

Synthesis of compound **6** was carried out as described previously[2]. For characterization of compounds, HPLC equipment (HP-Agilent 1100 with Diode Array detector), HRMS (LTQ Orbitrap XL mass-spectrometer, Thermo Fisher Scientific), and NMR (Bruker Avance-III 700 MHz NMR spectrometer magnetic field 16.4 T) were used.

Synthesis of compound 3

The starting compound amrubicinone (**1**) and 1,4-di-*O*-acetyl-*N*-trifluoroacetyl-β-L-daunosamine (**2**) (2 eq) were dissolved in dry THF under an argon atmosphere. 4Å molecular sieves were added, followed by dropwise addition of diethyl ether to the stirred reaction mixture. The reaction mixture was cooled to -33ºC and trimethylsilyl trifluoromethanesulfonate (2 eq) was added. After addition of trimethylsilyl trifluoromethanesulfonate (TMSOTf), the reaction mixture was stirred for 1.5h at -33ºC followed by stirring at -25…-20 ºC. After the starting material was consumed, the reaction mixture was poured into a pre-cooled mixture of saturated $NaHCO_3$ aq. solution and ethyl acetate. The phases were separated, aqueous phase was extracted once with ethyl acetate. The organic phases were combined, washed with brine and dried over Na_2SO_4. The solvent was evaporated and crude product was purified by column chromatography on silica gel (eluent: diethyl ether/ethyl acetate). The compound **3** was obtained with 90% of yield. The product was not 100% pure according to NMR (Figure S3), but was used in next step as such.

Synthesis of compound 4

Compound **3** was dissolved in dry ethanol and $NaHB(OAc)_3$ (2.1 eq) was added. The reaction mixture was stirred for 1h, and the consumption of the starting material was confirmed by TLC. The solvent was evaporated and water was added to the residue. The target product was extracted with diethyl ether, the solvent was evaporated from combined extracts under reduced pressure and the crude product was purified by column chromatography on silica gel (eluent DCM/MeOH 19/1). Compound **4** was obtained with 26% of yield and was characterized by ESI-MS (calculated [M+H] 637.2, found 637.2) (Figure S4).

Synthesis of compound 5

The compound **4** was dissolved in a mixture of tetrahydrofuran/methanol/water 2/2/1 and LiOH*H_2O (10 eq) was added at 0 °C. The reaction mixture was stirred for 20 h at 4 °C. After this period, water was added to the violet-colored mixture and the mixture was basified until pH 8.2 by dropwise addition of HCl 10% aqueous solution. The target product was extracted with chloroform. The combined organic extracts were dried with Na_2SO_4 and the solvent was evaporated under reduced pressure. The crude product was purified by column chromatography using lower phase of chloroform/methanol/aqueous ammonia mixture and chloroform as eluents. Compound **5** was obtained with 56% of yield after purification and characterized by 1H and 13C-NMR (Figure S5, S6).

Synthesis of compound 6

The obtained purified compound **5** was reacted with 1.9 eq of paraformaldehyde in dry chloroform for 3 days, the unreacted compound **5** was separated by filtration through 0.45 µm pore filter, the obtained solution was concentrated and triturated with diethyl ether to obtain compound **6**. The product is considered a key intermediate in synthesis and structure was proven by NMR (Figure S7-S10).

Synthesis of compound 8

Compound **6** (100 mg, 0.19 mmol) was dissolved in 6 mL of dry dimethyl formamide and of 4-nitrophenyl-(acetyloxy)-methylcarbonate (49 mg, 1 eq, 0.19 mmol) was added. The mixture was stirred for 26 h at room temperature under argon atmosphere. Under these conditions, the compound **7** is formed, and only the oxazolidine cycle on sugar moiety is available for reaction (the nitrogen atom at C9 is strongly sterically hindered). After this period, reaction mixture was partially concentrated (to ~1 mL) at room temperature under reduced pressure, mixed with 6 mL of solution of acetic acid (1%) in acetonitrile:water (1:1) and stirred for 2 h to hydrolyze the unreacted oxazolidine cycle in intermediate compound **7**. The obtained mixture was purified by preparative HPLC (Column Luna C18(2) Axia 27.2x250 mm, eluent system water/acetonitrile) (Figure S15). A total of 22 mg of conjugate **8** was separated. The product was characterized by NMR (Figure S11-S14) and High Resolution Mass Spectrometry (HRMS, calculated MW 627.2185, found MW 627.2182, Figure S16).

Synthesis of Fluorescein-PEG-PCL (FAM-PEG-PCL)

Mal-PEG-PCL (20 mg) was dissolved in 0.3 mL of nitrogen-purged DMF and 2 eq of FAM-Cys dissolved in 0.1 mL of nitrogen-purged DMF were added to the solution. The mixture was stirred overnight at RT. The solution was diluted in 2 mL of mQ water and dialyzed against water using a dialysis cassette (Thermo Scientific, USA) with a MW cut-off of 10 KDa to remove the excess of FAM-Cys. The resulting suspension was freeze-dried and a yellow powder was obtained.

Synthesis of peptide-functionalized polymersomes (PS)

PS were prepared using the film hydration method, following a protocol optimized from a previous work[3]. For the optimization of the RPARPAR peptide density on the surface of PS, FAM-PEG-PCL, mal-PEG-PCL, and PEG-PCL were mixed and dissolved in 0.5 mL acetone. The total amount of polymer was 5 mg. Different percentages of mal-PEG-PCL were used (0; 2; 5; 10; and 20%), and all the PS samples contained 5% of FAM-PEG-PCL polymer. The acetone was evaporated with nitrogen flow forming a thin polymeric film on the wall of the glass vial (Sigma-Aldrich, Germany). Next, the film was hydrated with 0.4 mL of PBS pH 7.4 previously purged with nitrogen flow, heated for 30 seconds in 65°C water bath and sonicated for 30 seconds. The heating and sonication steps were repeated until the PS were formed and polymer aggregates were not observed in the suspension. After that, 4 equivalents of Cys-RPAR peptide respect the mal-PEG-PCL were dissolved in 0.1 mL of PBS and added to the suspension of formed PS. The sample was sonicated for additional 10 minutes, mixed in the shaker for 3 h at RT and kept overnight at 4°C. The final volume of PS samples was 0.5 mL and total polymer concentration 10 mg/mL.
For the formation of FAM-labeled LinTT1-targeted PS (FAM-LinTT1-PS**)**, 1 mg of mal-PEG-PCL and 4 mg of PEG-PCL were used to form the polymeric film. The PS were formed as described above and 4 equivalents of FAM-Cys-LinTT1 peptide respect the mal-PEG-PCL were used for conjugation.
For the synthesis of non-targeted PS, only the PEG-PCL copolymer was used.

Nanoencapsulation of UTO

For the encapsulation of UTO inside PS, 50 nmoles of UTO were dissolved in 100 µL of acetone and added to the polymers dissolved in acetone (total amount of polymer was 5 mg). The acetone was evaporated to form the polymer/drug film and the PS were formed as described above.

Nanoencapsulation of Doxorubicin (DOX)

For DOX encapsulation inside PS, the polymeric film was hydrated with 1 mM DOX solution in PBS pH 7.4 and the PS were formed as described above.

Synthesis of DiR-labelled PS

For the encapsulation of DiR dye, 25 µg of DiR (5 µL of a solution 5 mg/mL in acetone) were added to the polymers dissolved in acetone (total amount of polymer was 5 mg). The acetone was evaporated to form the polymer/dye film and the PS were formed as described above. The final amount of DiR in the PS sample was 0.5% w/w.

Purification of PS

PS samples were purified by size exclusion chromatography. Agarose beads with a diameter of 45-165 µm (Sephadex 4B gel) were used as stationary phase. The height of the Sephadex gel in a column was 8 cm. The PS sample was eluted with PBS pH 7.4.
After purification, the DiR-labeled PS were concentrated to 20 mg polymer/mL using Amicon Ultra centrifugal filters (0.5 mL, MW cut-off of 100 kDa). The filters containing the samples were centrifuged for 10 min at 13,000 g, and the PS solution retained in the filter was recovered.

Characterization of PS

For transmission electron microscopy (TEM), PS samples were diluted in mQ water (0.5 mg/mL) and transferred onto copper grids for 1 min, stained with 0.75% phosphotungstic acid (pH 7) for 20 sec, air-dried, and visualized using Tecnai 10 TEM (Philips, Netherlands). The average hydrodynamic diameter of PS was measured with dynamic light scattering (DLS) by using Zetasizer Nano ZSP (Malvern, USA). PS samples were diluted with PBS pH 7.4 until 1 mg/mL. Samples were scanned for 10 seconds at 173° and average over 10 runs. Zeta potential was measured using Zetasizer Nano ZSP (Malvern, USA) at 0.2 mg of polymer/mL in NaCl 10mM, performing 50 runs per sample.
The amount of encapsulated UTO and DOX was quantified using Nanodrop 2000c UV-VIS spectrophotometer (Thermo Scientific, USA). For UTO quantification, serial dilutions of UTO in methanol:water 1:1 were prepared and the absorbance at 490 nm was measured. For DOX quantification, serial dilutions of DOX in PBS were prepared and the absorbance at 490 nm was measured. Using gathered data, the linear trend line was constructed in MS Excel program and the formula was further used to evaluate the concentration of UTO and DOX inside PS. The drug-PS samples were measured at the absorbance of 490 nm.
To ensure the same amount of encapsulated DiR inside all PS samples, the absorbance of DiR at 756 nm was measured using the Nanodrop.

Efficiency of the peptide conjugation to PS

The percentage of FAM-PEG-PCL in the PS samples was quantified by fluorimetry. First, a calibration curve of FAM-Cys was prepared in DMSO:PBS 1:1 and the fluorescence at 480 nm/535 nm was measured using Victor X5 Multilabel Microplate Reader (Perkin Elmer, USA). PS samples (25 µL) were mixed with 25 µL of MeOH and the fluorescence was measured to calculate the percentage of FAM-PEG-PCL in the PS composition.
To estimate the amount of peptide on the PS with optimum peptide density, PS were formed using 20% of Mal-PEG-PCL and 80% of PEG-PCL and FAM-Cys-RPAR peptide was conjugated to the PS as described above. The standard curve of FAM-Cys-RPAR was prepared in PBS and the FAM fluorescence was measured by fluorimetry at 480 nm/535 nm. PS functionalized with FAM-Cys-RPAR peptide (25 µL) were mixed with 25 µL of DMSO and the fluorescence was measured to calculate the percentage of FAM-RPAR-PEG-PCL in the PS composition. Figure S18A and B shows the standard curves of FAM-Cys and FAM-Cys-RPAR, and the measurement of the fluorescence of the PS samples. The percentage of FAM-PEG-PCL in the PS composition was 4.9±0.3 and the percentage of FAM-peptide-PEG-PCL respect the total amount of polymer was 6% (Figure S18C).

Cell culture

PPC-1 human primary prostate cancer and MCF10CA1a human triple negative breast cancer cells were gathered from Erkki Ruoslahti laboratory at Cancer Research Center Sanford-Burnham-Prebys Medical Discovery Institute. M21 human melanoma cells were gathered from David Cheresh at University of California San Diego. Cells were cultured in DMEM medium containing 100 IU/mL of streptomycin, penicillin, and 10% FBS in 37°C incubator with 5% CO_2. Cells were detached using enzyme-free dissociation buffer (CellStripper, Fisher Scientific). When seeding certain number of cells was needed, the cells were mixed with 0.4% Trypan Blue and counted using Bio-Rad TC 10 automated cell counter (Bio-Rad, USA).

Cellular binding assessment by flow cytometry

PPC-1 and M21 cells were seeded in 24-well plates (100,000 cells/well; 0.5 mL/well) and let to grow for 24 h. Then the cells were incubated with the FAM-labelled PS samples (1 mg/mL) for 1 h, washed with PBS, detached, and transferred to 1.5 mL tubes. Next, the cells were washed 3 times with medium and resuspended in 0.3 mL of PBS. The samples were analyzed using flow cytometer (BD Accuri C6 Plus, BD Biosciences) and the data analysis was done using the same program.

Cellular uptake assessment by fluorescence confocal microscopy

PPC-1 cells and M21 cells (50,000 cells/well; 0.5 mL/well) were seeded in 24 well-plates (Croning, Sigma-Aldrich) with coverslips (12 mm diameter, Marienfeld-Superior) and let to grow for 1 day in 37°C incubator with 5% CO_2. Then the cells were incubated with FAM-labeled PS samples (1 mg/mL) for 1 h, washed thrice with 1 mL PBS, fixed with 4% PFA in PBS for 10 min at RT, and stained with 1 µg/mL of DAPI for 5 min at RT. The coverslips with attached cells were mounted with 20 µL of mounting medium (Fluoromount-G; Electron Microscopy Sciences) and sealed with nail polish. Confocal microscope FV1200MPE (Olympus, Japan) was used for cell imaging using UPlanSApo 60x/1.35na or 10x/0.4na objectives (Olympus, Japan). The images were analyzed using Olympus FluoView Ver.4.2a Viewer program.

Assessment of in vitro cytotoxicity

PPC-1 and M21 cells (10,000 cells/well) were seeded in 96-well plates and grown for 1 day at 37°C incubator with 5% CO_2. Dilutions of PS samples and free DOX were prepared in PBS. Free UTO was dissolved in DMSO and diluted in PBS until the final concentration of DMSO in the well was lower than 0.5%. Samples were added to the cells, incubated for 30 min, washed with 0.2 mL PBS and fresh medium was added. Cells were grown for additional 48 h. Then, 0.1 mL of MTT reagent (5 mg/mL in PBS) was added to the cells. After 2 h incubation in 37°C incubator with 5% CO_2, the MTT reagent was aspirated and the formazan crystals were dissolved in 0.1 mL of isopropanol. The absorbance was measured at 570 nm using Tecan Sunrise microplate reader (Tecan, Switzerland).
The evaluation of the IC_{50} of UTO and DOX was performed by Selvita (Poland). A panel of cultured tumor cells were seeded in an appropriate density for each cell line: U937 (30,000 cells/well), Jurkat E6.1 (80,000 cells/well), A549 (2,000 cells/well), and HT-29 (6,000 cells/well), on 96-well plates and treated with UTO at eight different concentrations (in culture medium with FBS) for both 30 min and 90 min (3 wells per condition). After treatment, cell cultures were carried out for further 24 and 72 h in fresh culture medium - respectively for cells growing in suspensions and adherent cells at 37°C, in a humidified atmosphere containing 5% CO_2. Solvent controls (H_2O or DMSO; 1%) were included in the test. After incubation, MTS/PMS reagent was added (for 1-2 h depending on cell line) and absorbance at 490 nm was recorded. A blank experiment detecting cell-free background absorbance was also performed in parallel.

Drug release study

To study the UTO cumulative release, UTO-loaded PS in PBS (0.25 mL) were incubated for different time periods (0; 1; 3; 24; and 48h) at 37°C and centrifuged using Amicon Ultra centrifugal filters (MWCO 100 kDa) for 20 min at 6,000 g at RT. The fluorescence of the filtrates was measured at 485 nm/535 nm using Victor X5 Multilabel Microplate Reader (Perkin Elmer, USA) to quantify the amount of released UTO (Figure S19).

In vivo experiments

The animal experiments were done accordingly to protocols approved by Estonian Ministry of Agriculture, Committee of Animal Experimentation (projects *#159* and *#160*). All the mice manipulations were done by the author.
For the MCF10CA1a orthotopic tumor induction, athymic nude female mice (*Hsd/Athymic Fox1 nu Harlan*; 7-8 weeks old) were injected in the mammary gland with $2x10^6$ MCF10CA1a cells in 50 µL of PBS. Tumors were developing during approx. 24 days until they reached 350 mm^3 in volume. The tumor volume calculations were obtained using the formula $V = (W^2 \times L)/2$ where *V* is tumor volume, *W* is tumor width, and *L* is tumor length[4].

Biodistribution of PS functionalized with Tumor Penetrating Peptides

Mice bearing orthotopic MCF10CA1a tumors were injected intravenously (i.v.) with targeted (LinTT1 or RPAR) and non-targeted DiR-labeled PS (80 mg/kg). The mice were anesthetized with 3% isoflurane (Forane, 99.9% w/w, Abbvie) blended with O_2 at 1 mL/min flow, and imaged using IVIS Spectrum In Vivo Imaging System (IVIS; PerkinElmer, US) at different time points (0; 1; 3; 6; 24; and 48 h post-injection). The parameters for *in vivo* imaging were as follows - exposure time: 0.5 sec; pixel binning: medium; F/stop: 4; excitation filter: 745 nm; emission filter: 830 nm; and stage temperature: 37°C. After the last imaging, the animals were sacrificed by perfusion with 10 mL PBS, the tumors and organs were excised and preserved in PFA 4% in PBS at 4°C. The signal from DiR-PS in tumor was measured selecting the region of interest (ROI) and quantified using Living Image 4.5.2. The area under the curve (AUC) in tumor was constructed from the *in vivo* quantification data.
For the ex vivo analysis of tumors treated with LinTT1-UTO-PS, UTO-PS and free DOX, the MCF10CA1a tumor-bearing mice were i.v injected with the PS samples at a dose of 5 mg of drug/kg. After 24h the mice were terminated, tumors extracted and the immunofluorescence microscopy was performed as described below.

Tissue immunofluorescence and confocal microscopy

PFA-fixed tissues were washed with PBS and left in PBS for 1 h at RT. Then the tissue was left overnight at 4°C in a solution of 15% sucrose in PBS and next day the solution was replaced with 30% sucrose solution in PBS. The tissues were frozen in freezing medium and cryosectioned to get 10 µm tissue sections. The sections were dried for 1 h at RT, permeabilized with PBS containing 0.2% Triton-X for 10 min, washed with PBS containing 0.05% Tween-20 (PBST), and blocked with PBST containing 5% BSA, 5% FBS, and 5% goat serum for 1 h. The tissues were incubated overnight at 4°C with rabbit anti-fluorescein (Thermo Fisher Scientific), rat anti-mouse CD31 (BD Biosciences), rat anti-mouse CD206 (Bio-Rad, USA), rabbit polyclonal anti-NRP-1, and rabbit anti-p32 (in-house) as primary antibodies in diluted blocking buffer (antibody dilution 1/100). Alexa 647-conjugated goat anti-rabbit IgG (Thermo Fischer Scientific) and Alexa 546-conjugated goat anti-rat IgG (Thermo Fischer Scientific) were used as secondary antibodies (antibody dilution 1/200). The sections were then washed with PBST and PBS and the nuclei were stained with 1 µg/mL DAPI in PBS for 10 min. Stained slides were mounted with mounting medium and sealed with nail polish. The tissues were imaged with fluorescence confocal microscope and the analysis of the images was performed as described above.

Statistical analysis

All of the statistical analyses were performed with Statistica 8 software (StatSoft, USA) using Fisher LSD and the one-way ANOVA tests. IC_{50} (half-maximal inhibitory concentration) was determined using log(inhibitor) versus response - Variable slope (four parameters) model (GraphPad Prism version 5.03 for Windows, GraphPad Software, La Jolla California USA).

Supplementary Figures

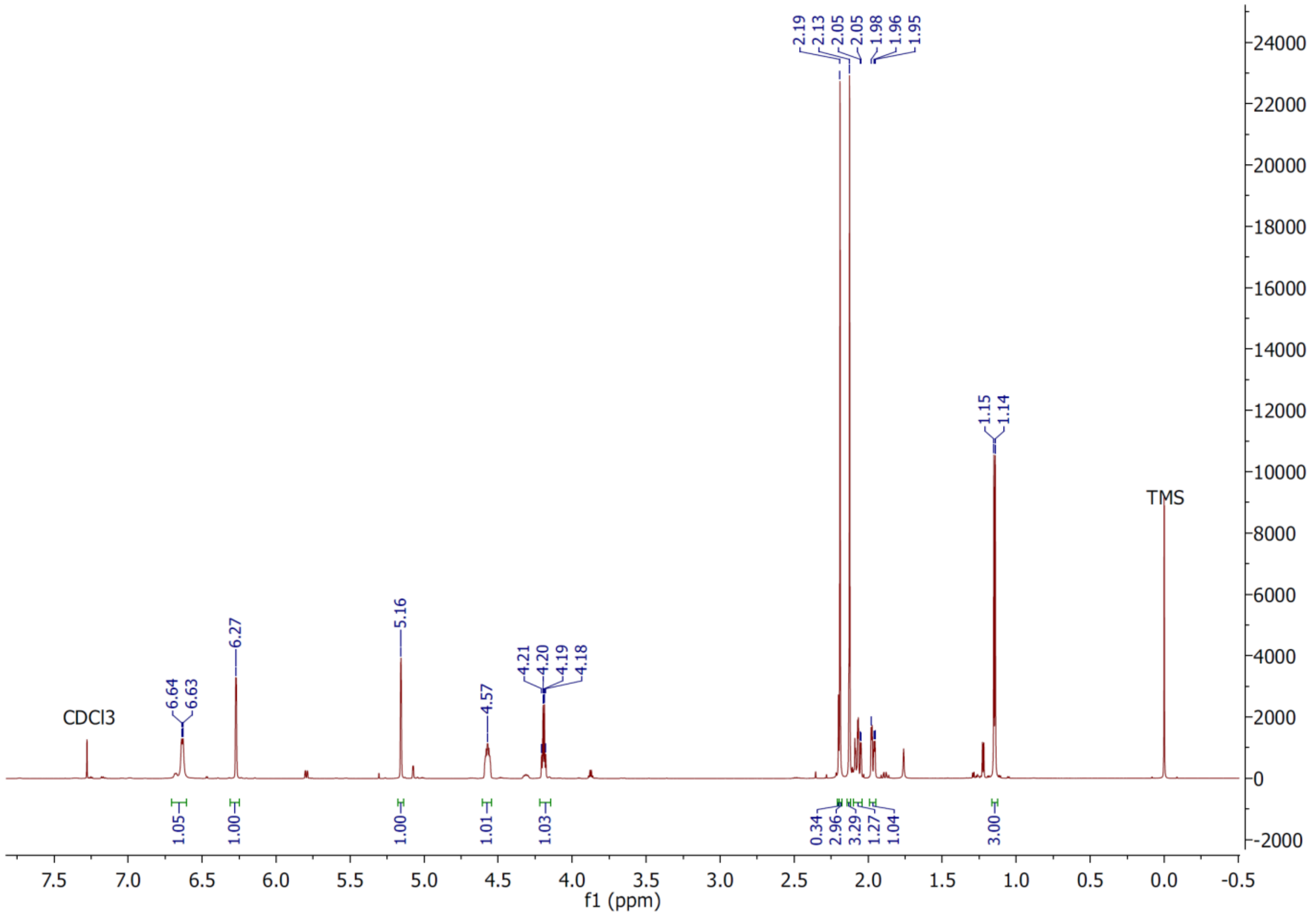

Figure S1. 1H-NMR spectrum of compound 2 measured in $CDCl_3$, 700 MHz.

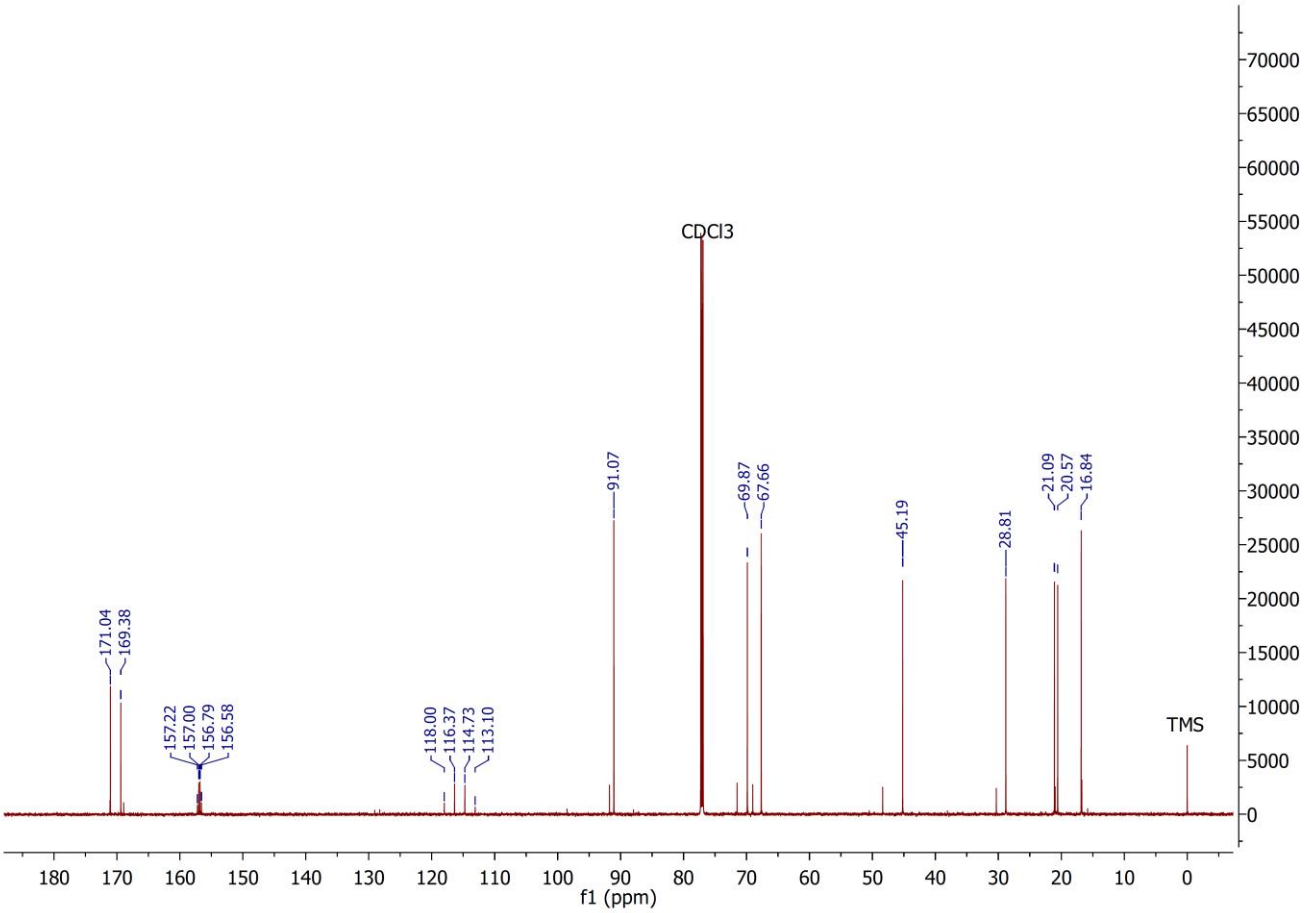

Figure S2. 13C-NMR spectrum of compound 2 measured in $CDCl_3$, 175 MHz.

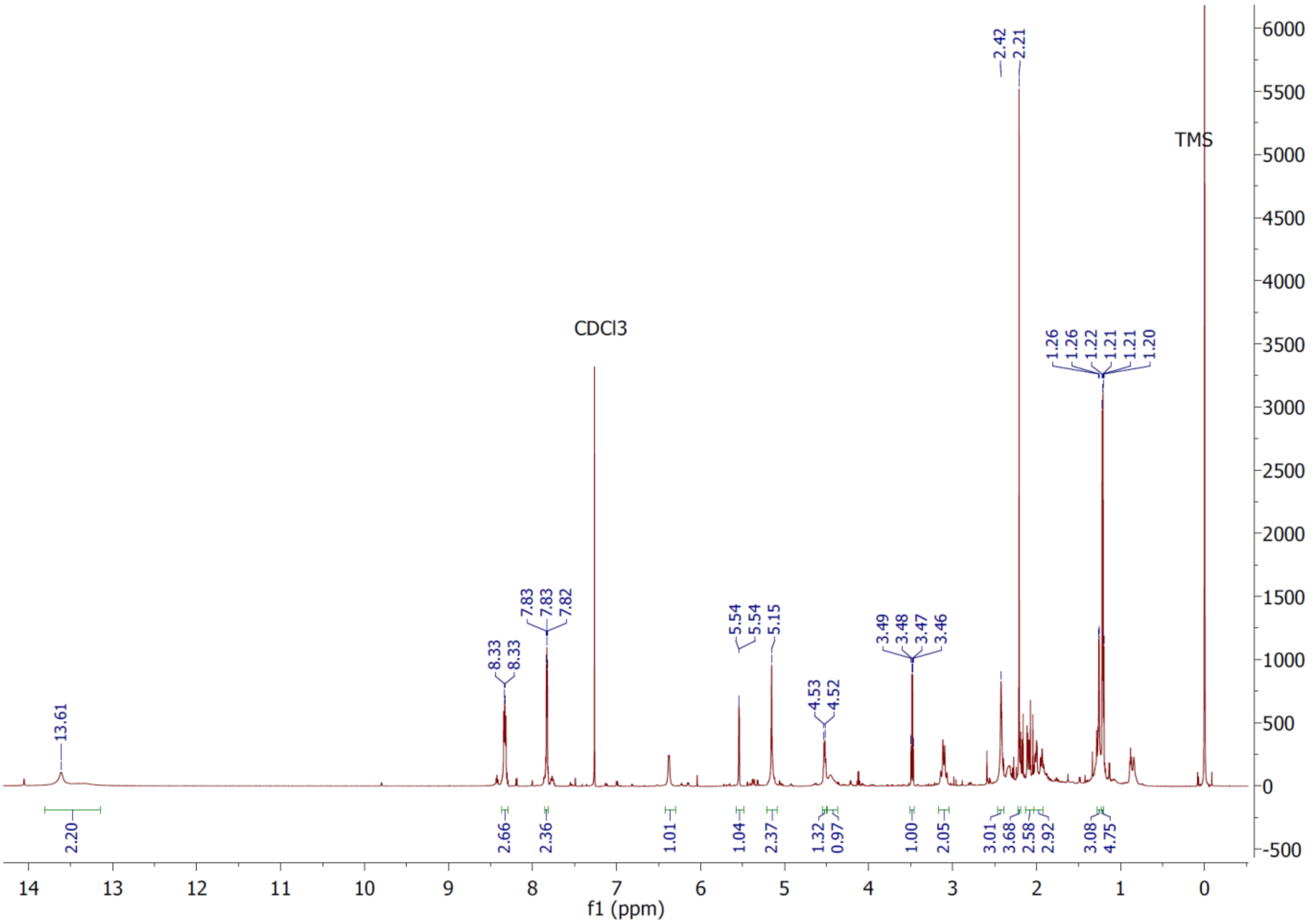

Figure S3. 1H-NMR spectrum of compound 3 measured in $CDCl_3$, 700 MHz.

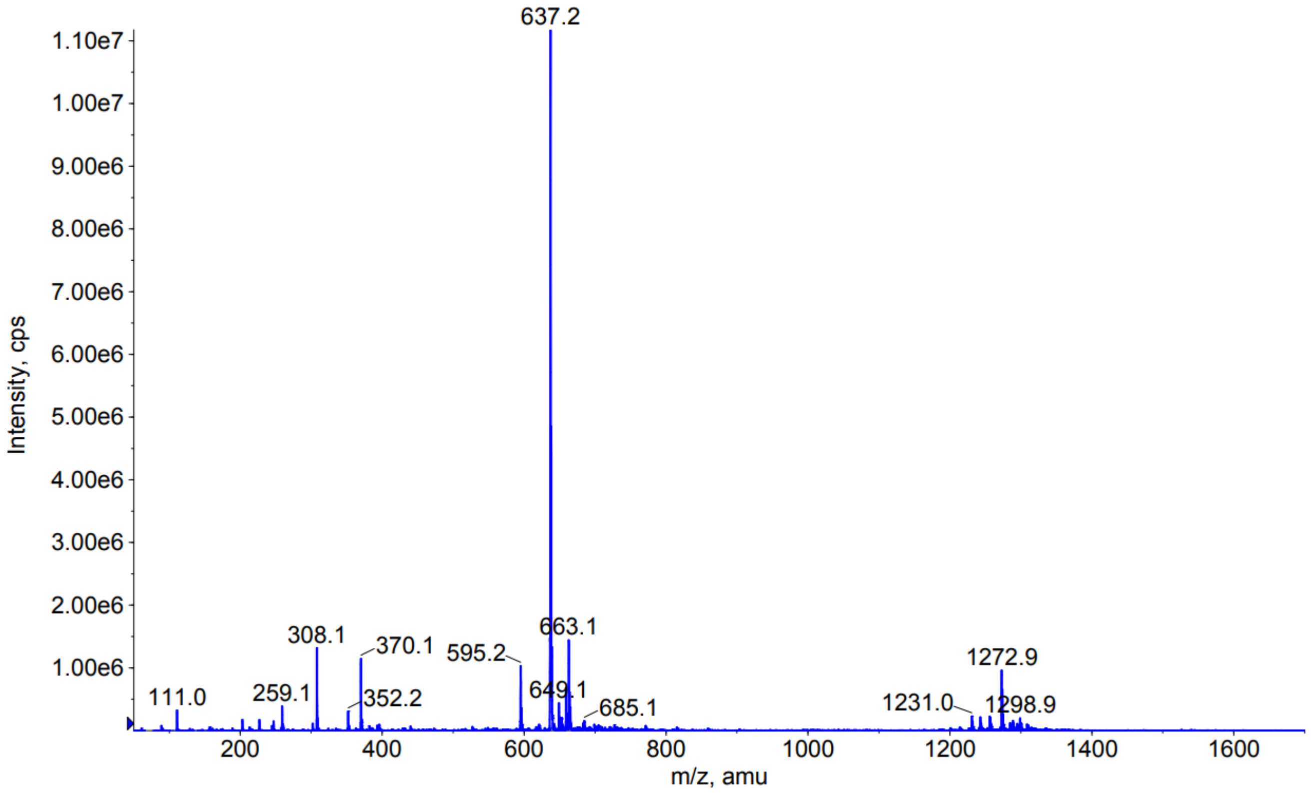

Figure S4. ESI-MS of compound 4. Spectrum recorded with Sciex API3200 QTrap mass spectrometer. Agilent 1260 series HPLC is used as injection pump.

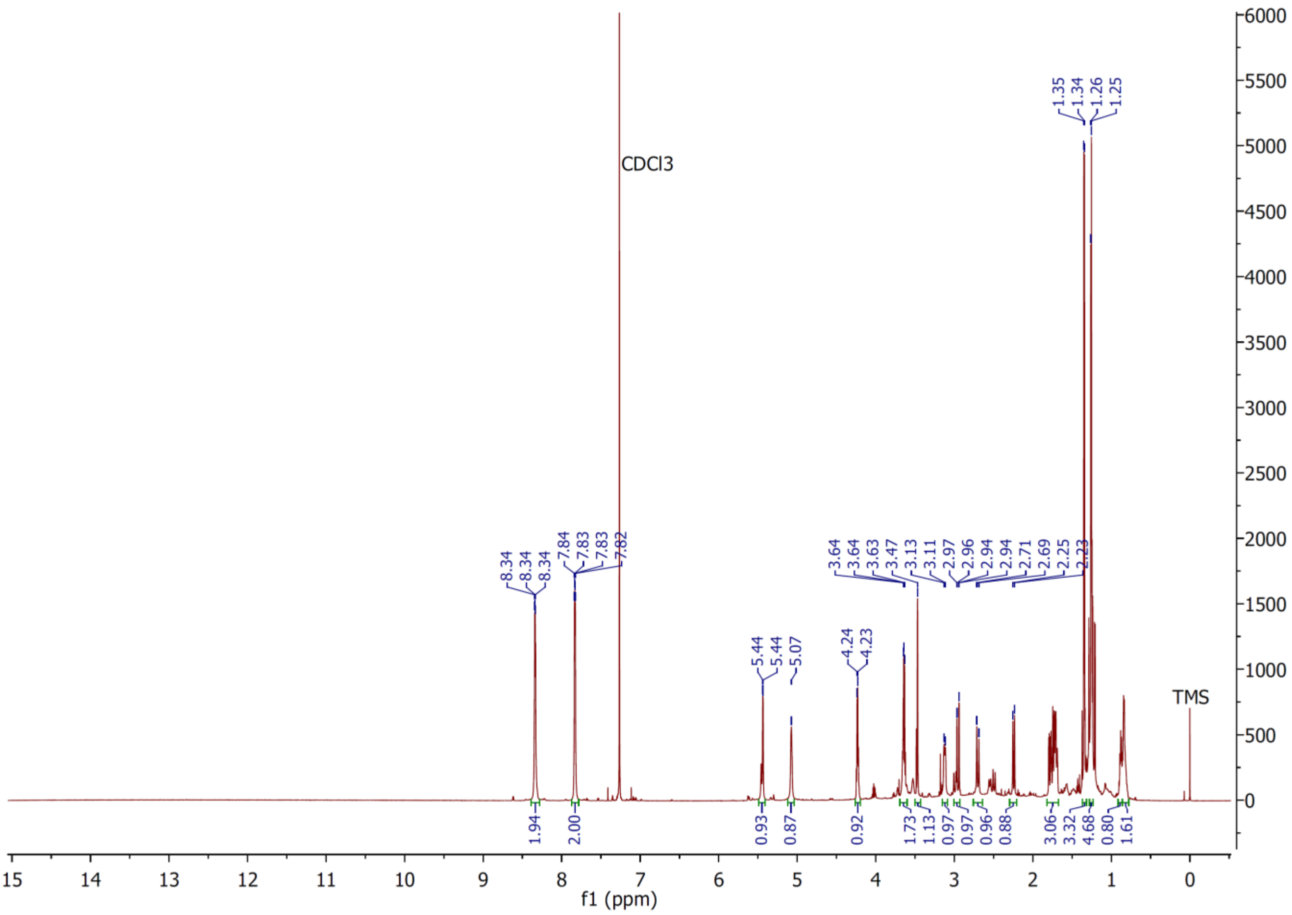

Figure S5. 1H-NMR spectrum of compound 5 measured in $CDCl_3$, 700 MHz.

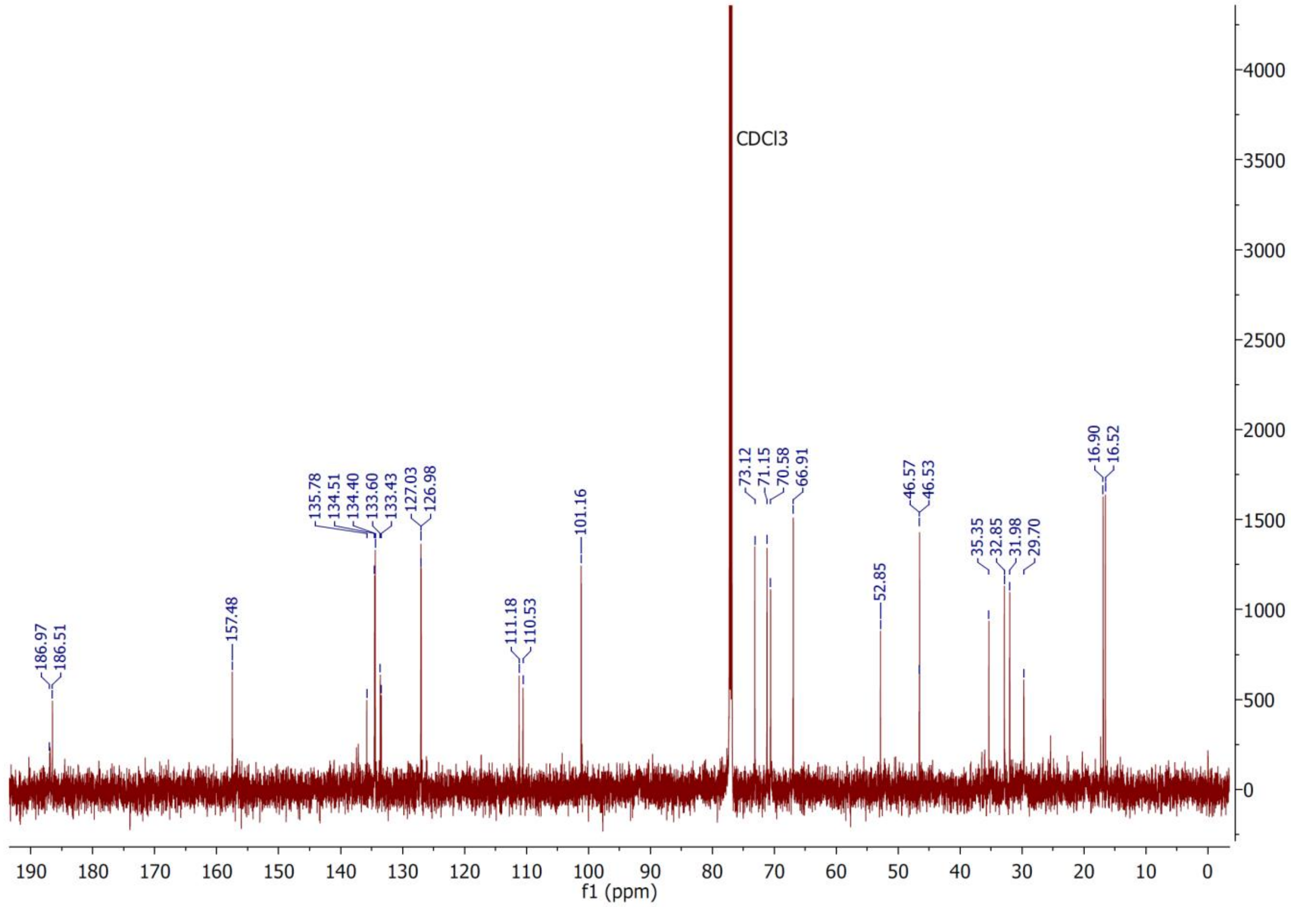

Figure S6. 13C-NMR spectrum of compound 5 measured in $CDCl_3$, 175 MHz.

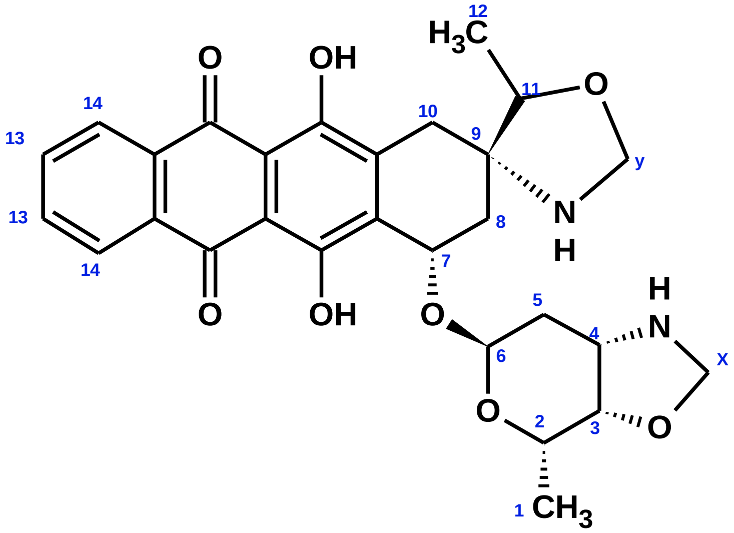

C number	13-C	1H
1	16.29; 16.37	1.31-1.34
2	65.72; 65.81	3.89-3.93
3	74.70; 74.73	3.73-3.75
4	53.82	3.52
5	28.25; 28.28	1,78; 2.38
6	99.09; 99.18	5.56
7	69.56; 69.65	5.10; 5.14
8	35.00; 37.76	1.89/2.26; 2.12
9	59.26; 59.63	-
10	27.00; 33.44	2.40/3.16; 2.35/3.27
11	80.25; 81.31	3.64; 3.79
12	15.40, 15.57	1.26-1.31
13		
14		
X	81.35	4.30/4.69
y	79.52; 79.76	4.31/4.68; 4.59/4.65

Figure S7. Assignment of NMR signals for compound 6. The carbon C11 is racemic (all others are stereochemically pure), thus the toxin is present in form of diastereomers. Therefore, there are two sets of signals, with the differences being bigger in anthracycline part and less in sugar part. The aromatic part has not been fully assigned. 13C-spectrum has one unidentified signal at 29.68 ppm (correlation to –CH2- type of protons at 1.24 ppm). 1H spectrum shows some impurities in aliphatic region (0.78-0.9 ppm), not giving correlations to any carbons.

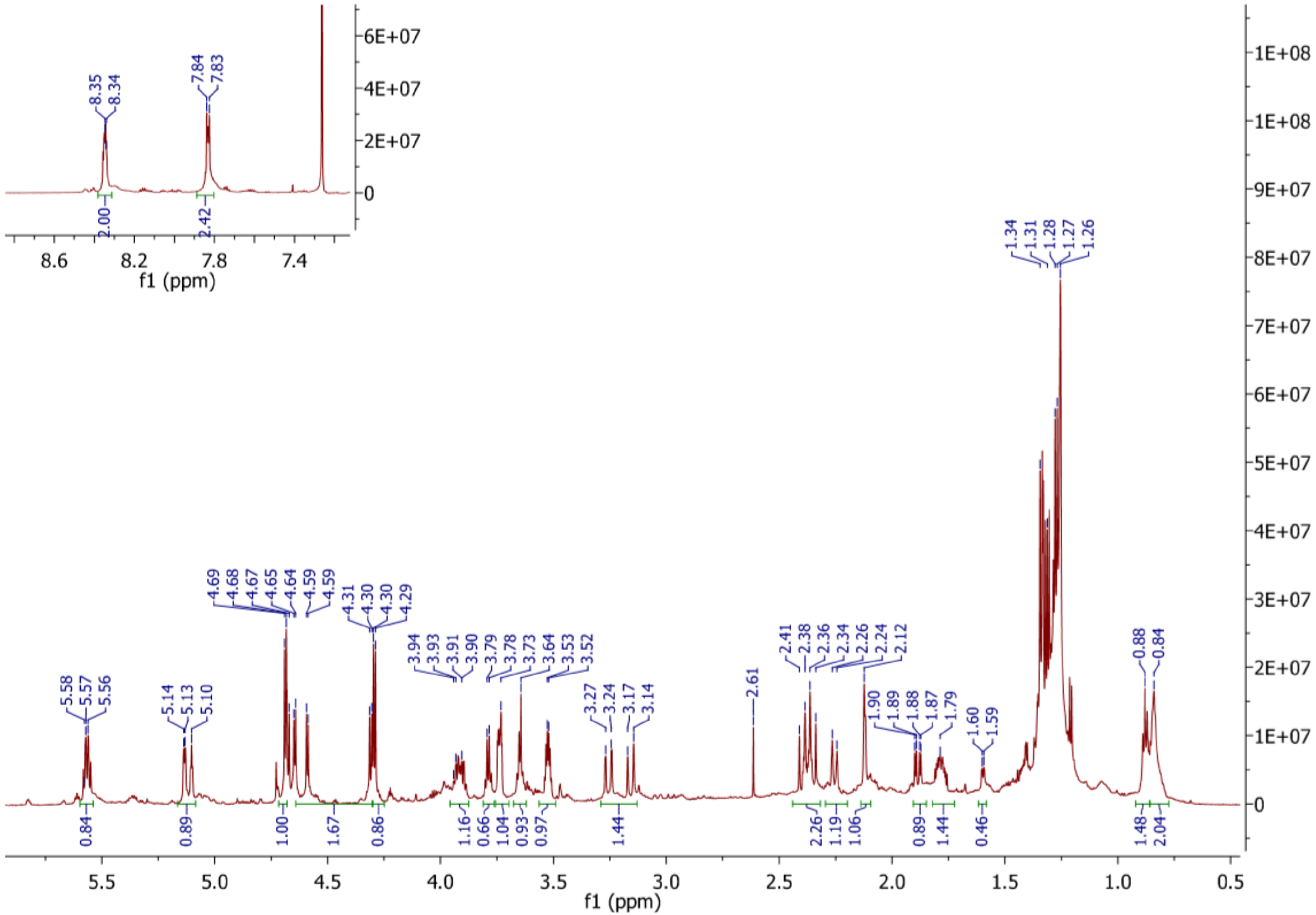

Figure S8. 1H-NMR of compound 6 measured in $CDCl_3$, 700 MHz.

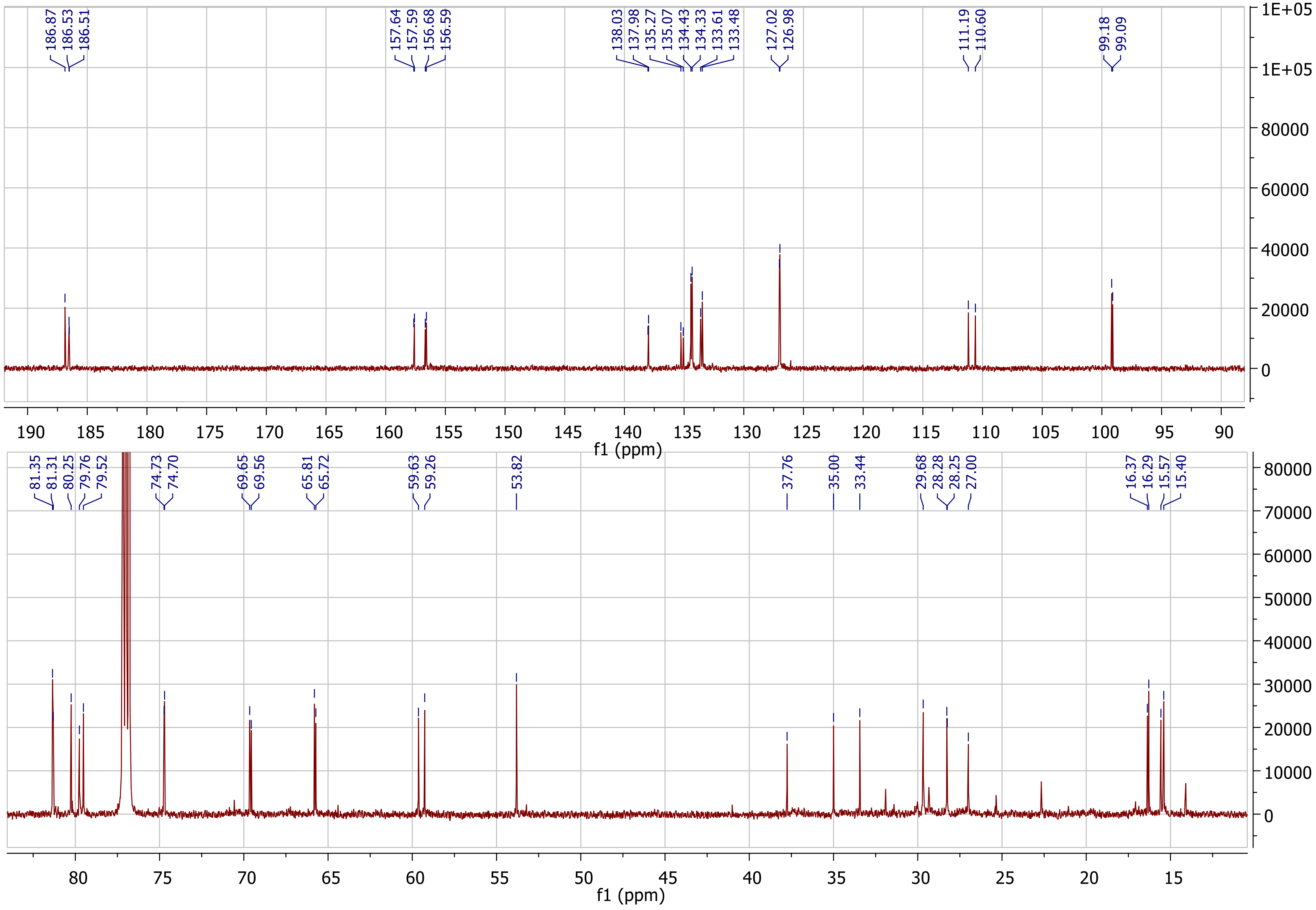

Figure S9. 13C-NMR of compound 6 measured in $CDCl_3$, 175 MHz.

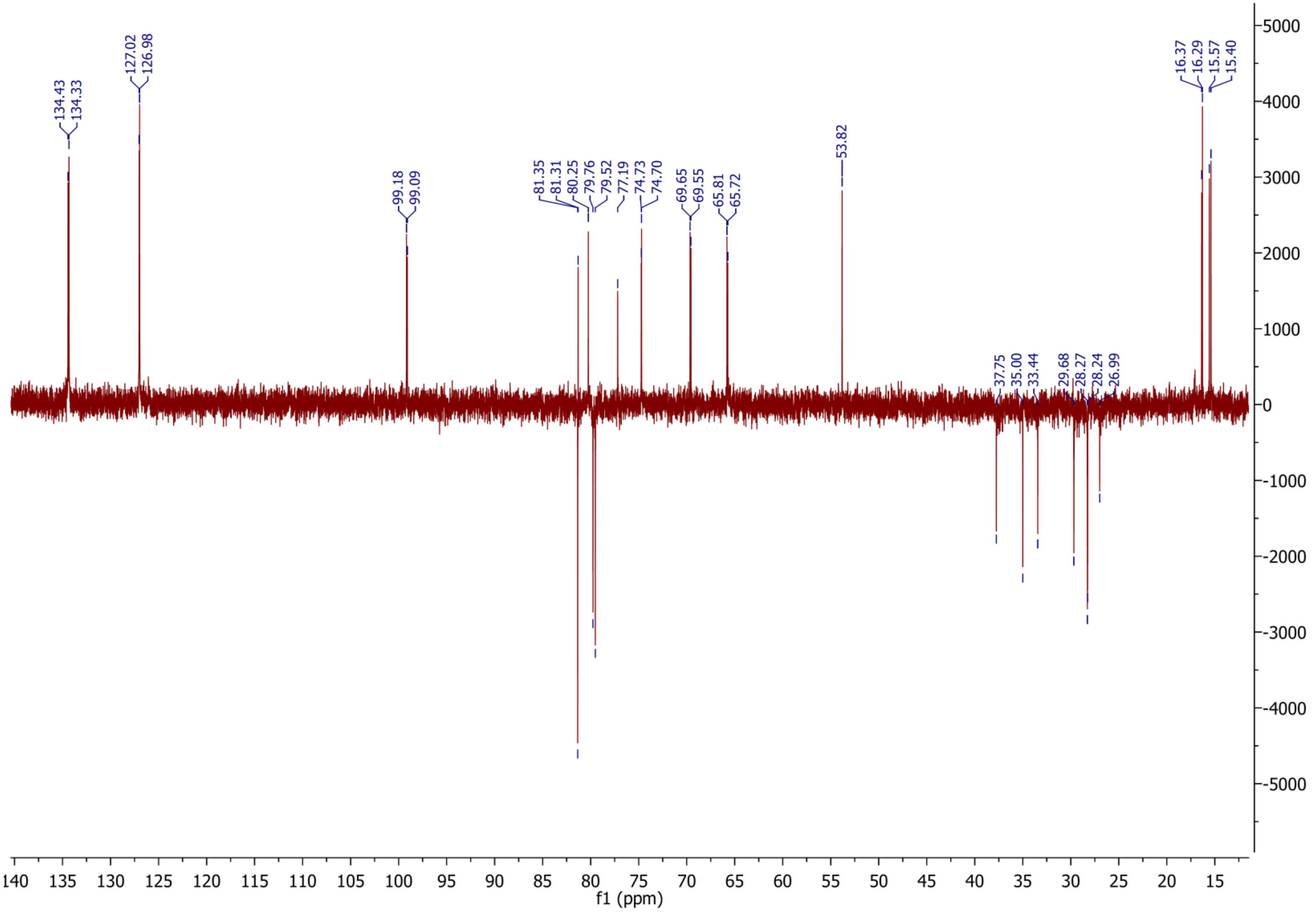

Figure S10. 13C-DEPT spectrum of compound 6 measured in $CDCl_3$, 175 MHz.

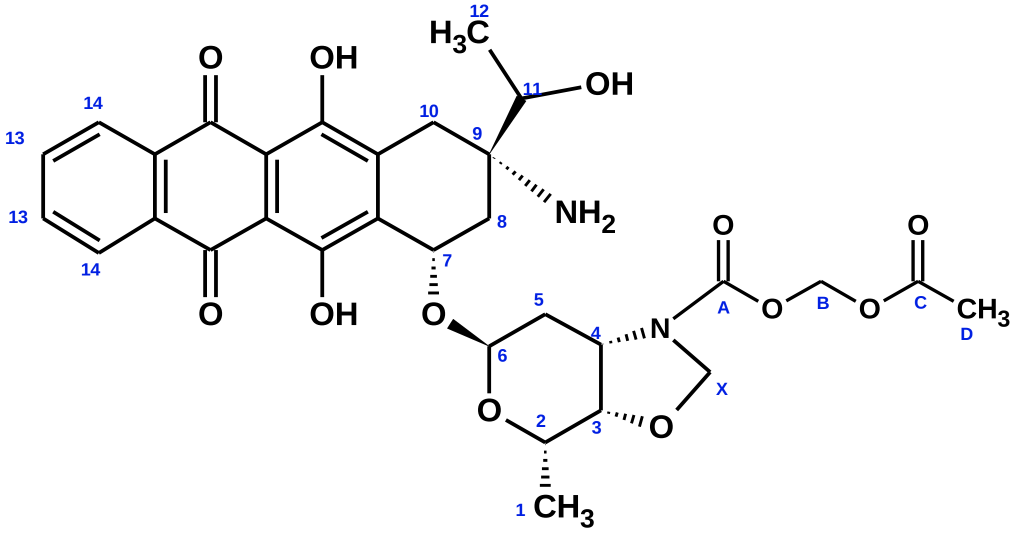

C number	13-C	1H
1	15.83-15.98	1.39 (3H)
2	65.33, 65.44	4.36-4.45 (1H)
3	78.03, 78.23	4.92-4.98 (1H)
4	49.86, 50.00	4.00-4.36 (1H)
5	28.59, 28.69	1.84-2.00 (1H), 2.12-2.22 (1H)
6	97.80-98.58	5.40-5.63 (1H)
7	64.77-65.10	5.40-5.63 (1H)
8	31.01, 33.89, 34.06	1.84-2.00 (1H), 2.44-2.63 (1H)
9	58.42-58.81	-
-NH_2 at C9	-	8.07-8.42 (2H)
10	27.03, 30.27	2.87-3.05 (1H), 3.41-3.60 (1H)
11	70.51-71.13	4.00-4.36 (1H)
-OH at C11	-	4.98-5.10 (1H)
12	17.00, 17.34	1.34 (3H)
13, 14	127.11, 127.19 / 134.77	7.80-7.89 (2H), 8.27-8.34 (2H)
Aromatic phenols	-	13.17-13.27 (1H), 13.52-13.60 (1H)
X	78.45, 78.54	4.00-4.36 (2H)
A	156.12, 156.18	-
B	79.95, 80.03	5.66-5.78 (2H)
C	169.70	-
D	20.73, 20.79	2.03-2.12 (3H)

Figure S11. Assignment of NMR signals for compound 8 (UTO)**.** The carbon C11 is racemic (all others are stereochemically pure), thus the toxin is present in form of diastereomers. Therefore, there are two sets of signals, with the differences being bigger in anthracycline part and less in sugar part. Additionally, the acetyloxy-methylcarbonate group seems to exhibit rotamerism, as can be seen from splitting of the proton signal of the carbon D. Therefore, most of the carbon signals are split and also exact alignment of proton multiplets is complicated. The aromatic part has not been fully assigned.

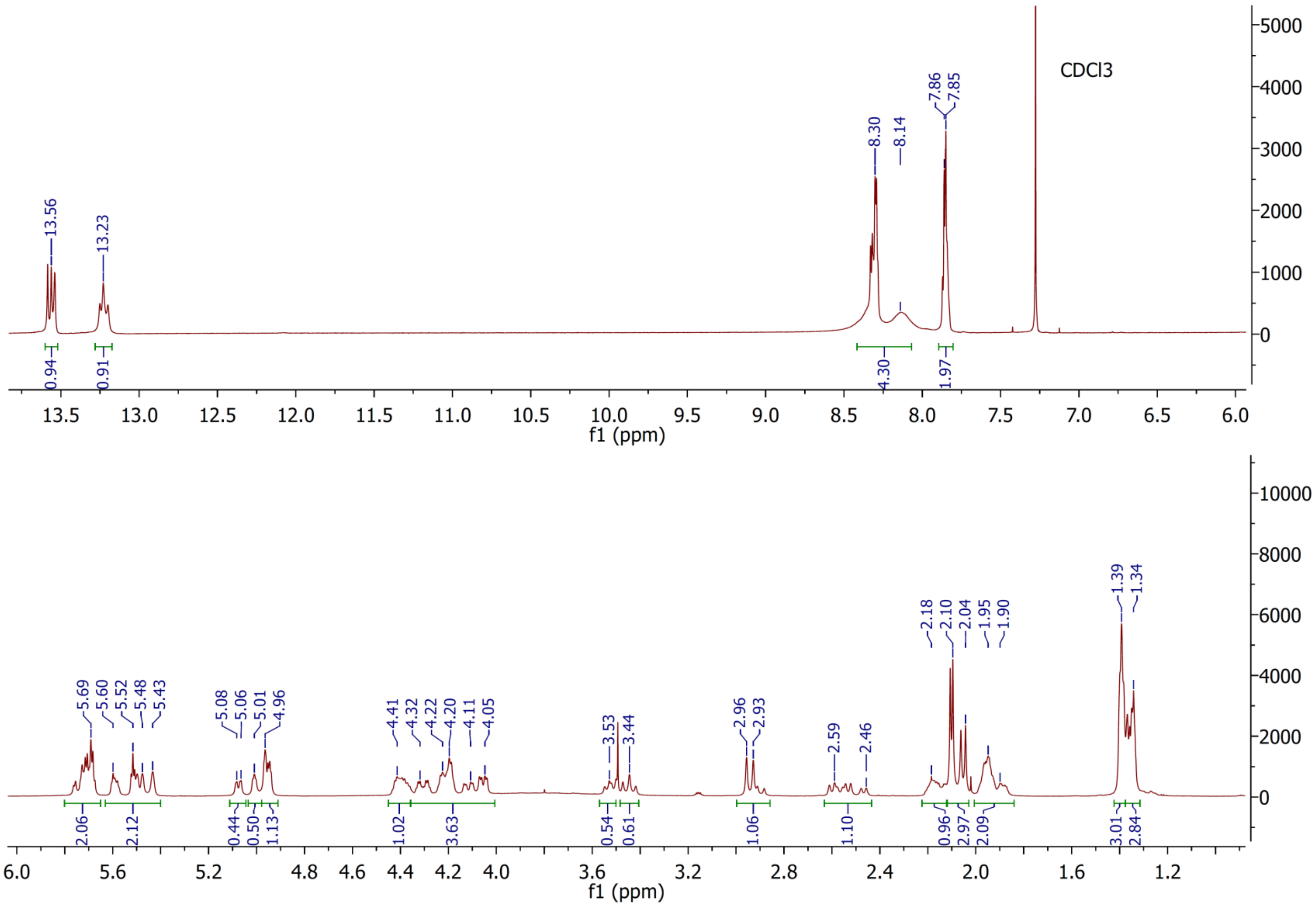

Figure S12. 1H-NMR spectrum of compound 8 (UTO) measured in $CDCl_3$, 700 MHz.

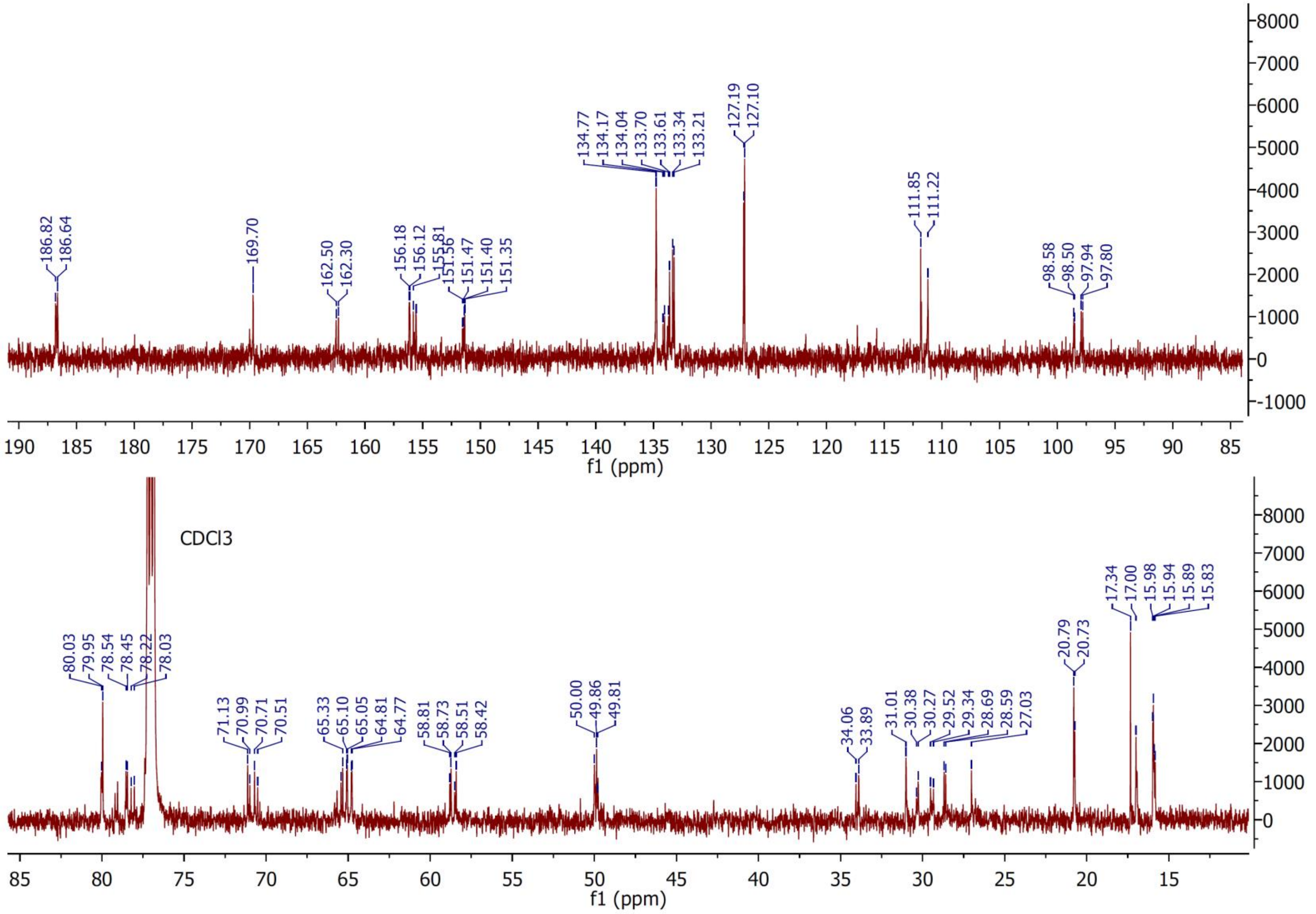

Figure S13. 13C-NMR spectrum of compound 8 (UTO) measured in $CDCl_3$, 175 MHz.

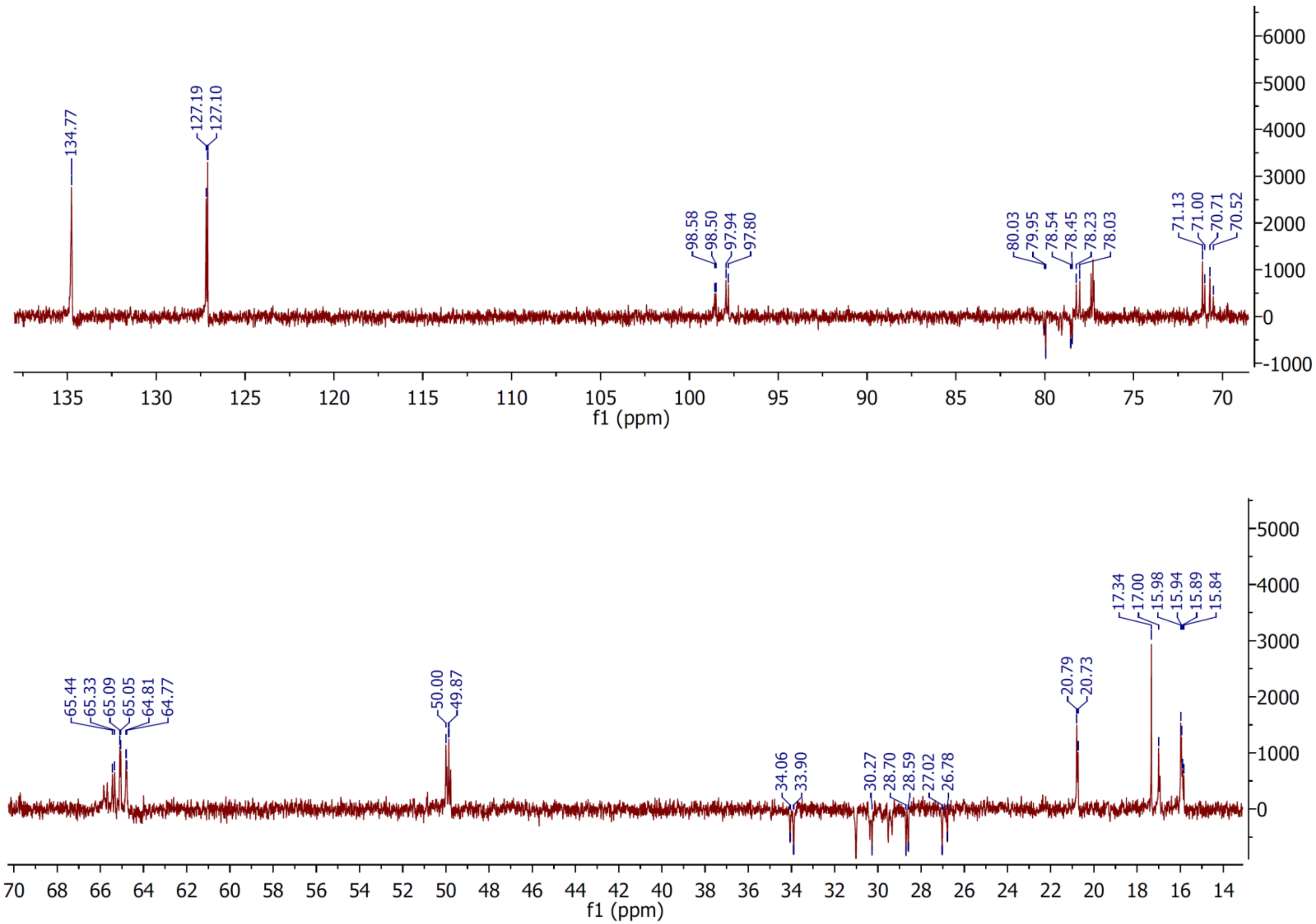

Figure S14. 13C-DEPT spectrum of compound 8 (UTO) measured in $CDCl_3$, 175 MHz.

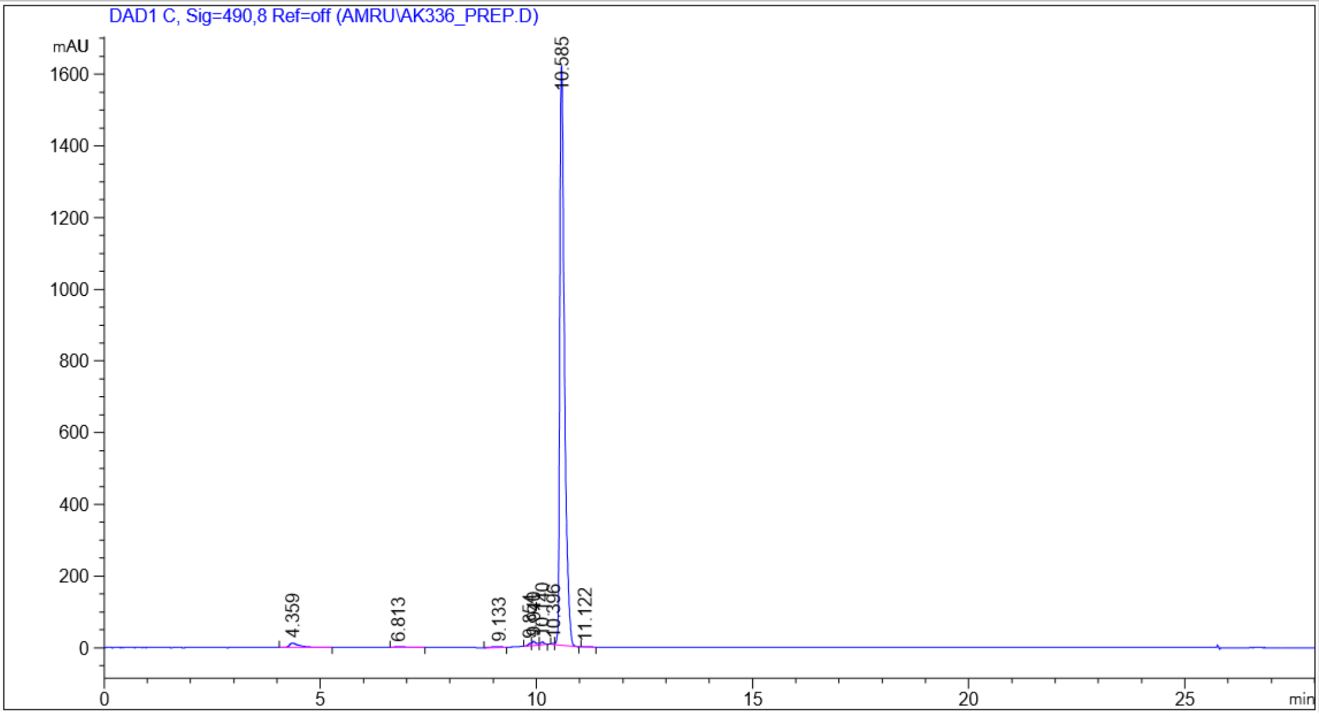

Figure S15. HPLC chromatogram of compound 8 (UTO). The conditions used for HPLC were the following: HP-Agilent 1100 equipment with DAD detector, wavelength 490 nm; column Phenomenex Luna C18(2) 250x4.6 mm, 5 µm; Column temperature 40° C; Eluents: water + 0.1% trifluoracetic acid/Acetonitrile + 0.1% trifluoroacetic acid; flow rate 1.2 mL/min; Gradient: 0-5 min 30% MeCN, 5-10 min 30%→80% MeCN; 10-18 min 80%→95% MeCN; 18-22 min 95% MeCN, 22-23 min 95%→30% MeCN, 23-28 min 30% MeCN.

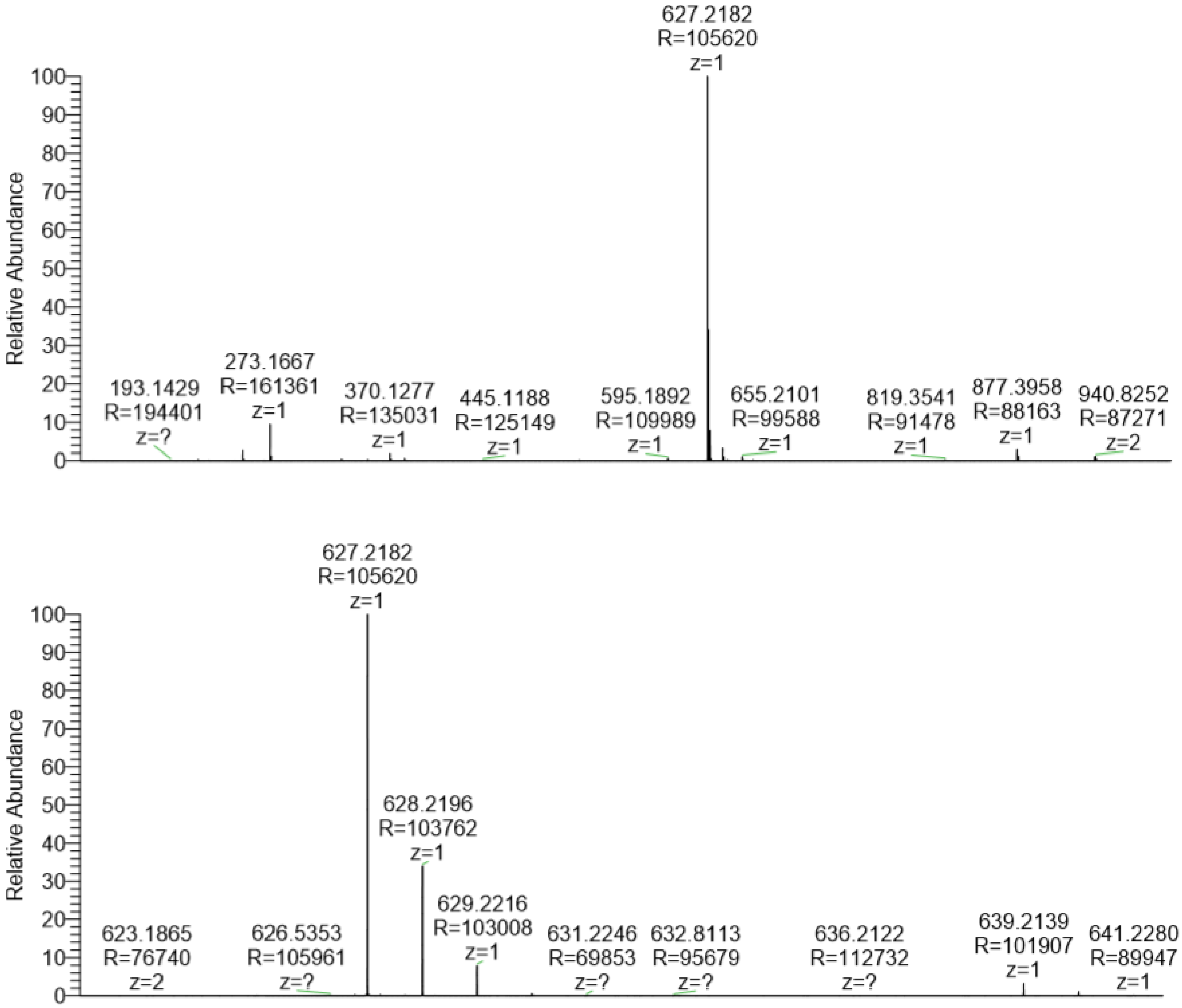

Figure S16. HRMS of compound 8 (UTO). Upper spectrum from m/z = 100 to1000, Lower spectrum is from m/z = 622 to 650.

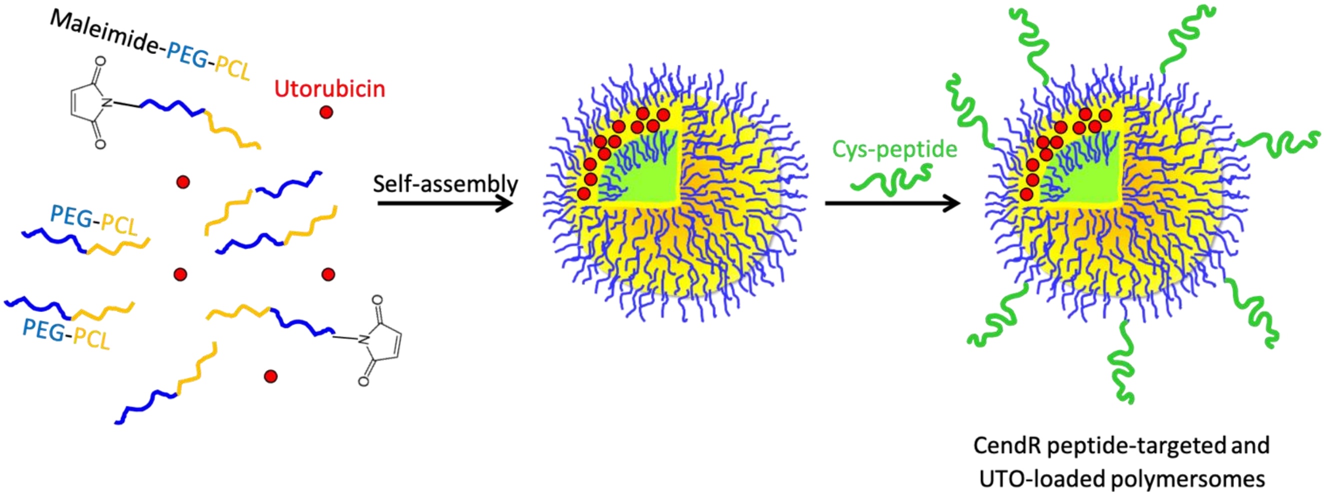

Figure S17. Illustration of the CendR peptide-targeted and UTO-loaded polymersome formation

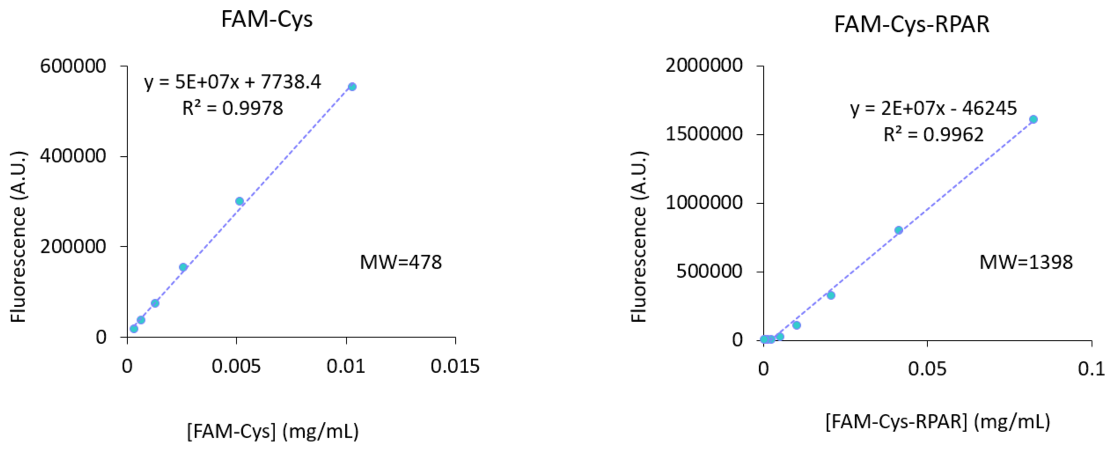

C

Sample	Fluorescence (A.U)	% FAM-PEG-PCL/polymer	% Peptide-PEG-PCL/polymer
RPAR-PS (20% Mal-PEG-PCL, 5% FAM-PEG-PCL)	300024	5.5	
RPAR-PS (10% Mal-PEG-PCL, 5% FAM-PEG-PCL)	258776	4.7	
RPAR-PS (5% Mal-PEG-PCL, 5% FAM-PEG-PCL)	244384	4.5	
RPAR-PS (2% Mal-PEG-PCL, 5% FAM-PEG-PCL)	225941	4.0	
RPAR-PS (0% Mal-PEG-PCL, 5% FAM-PEG-PCL)	317230	5.8	
FAM-RPAR-PS (20% Mal-PEG-PCL)	330620		6.1

Figure S18. Efficiency of peptide conjugation to PS. (A) and **(B)** Standard curves of FAM-Cys and FAM-Cys-RPAR respectively. **(C)** Table of the fluorescence of the samples in arbitrary units (A.U.) and % of FAM-PEG-PCL and peptide-PEG-PCL in PS samples.

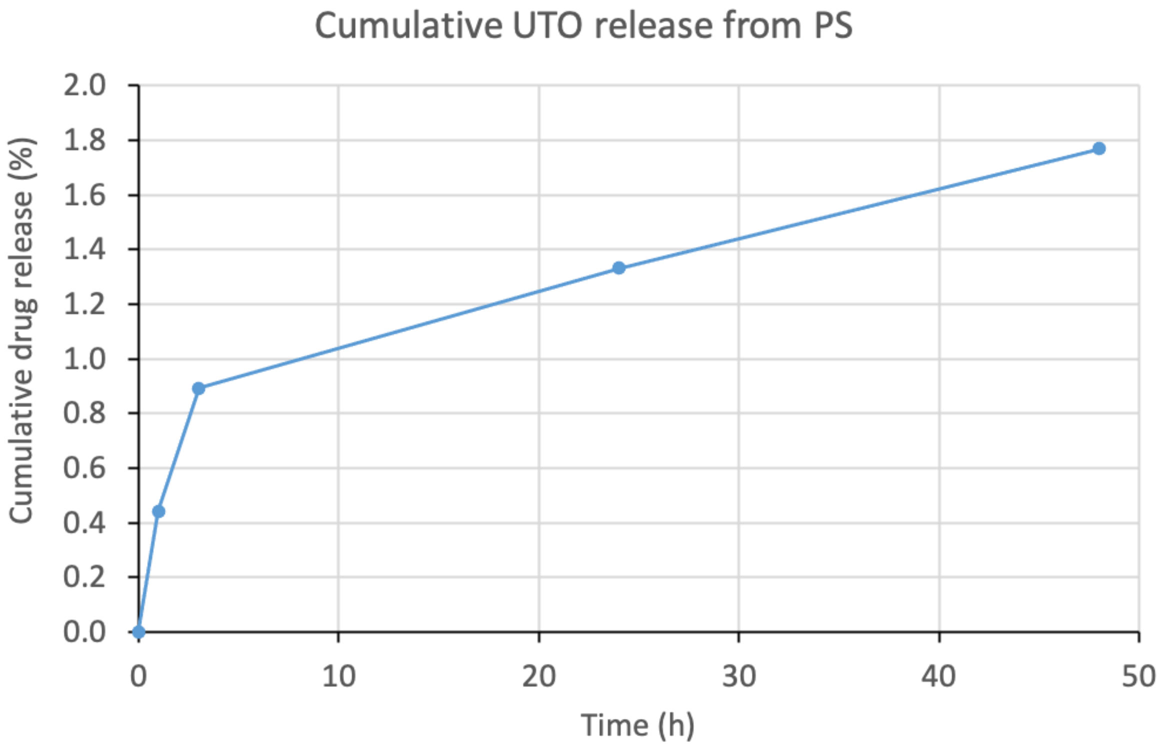

Figure S19. Cumulative UTO release from PS. UTO-loaded PS were incubated in PBS at 37°C, centrifuged using Amicon centrifugal filters (MWCO=100 KDa) and the fluorescence of the filtrates was measured at 485 nm/535 nm to quantify the released UTO.

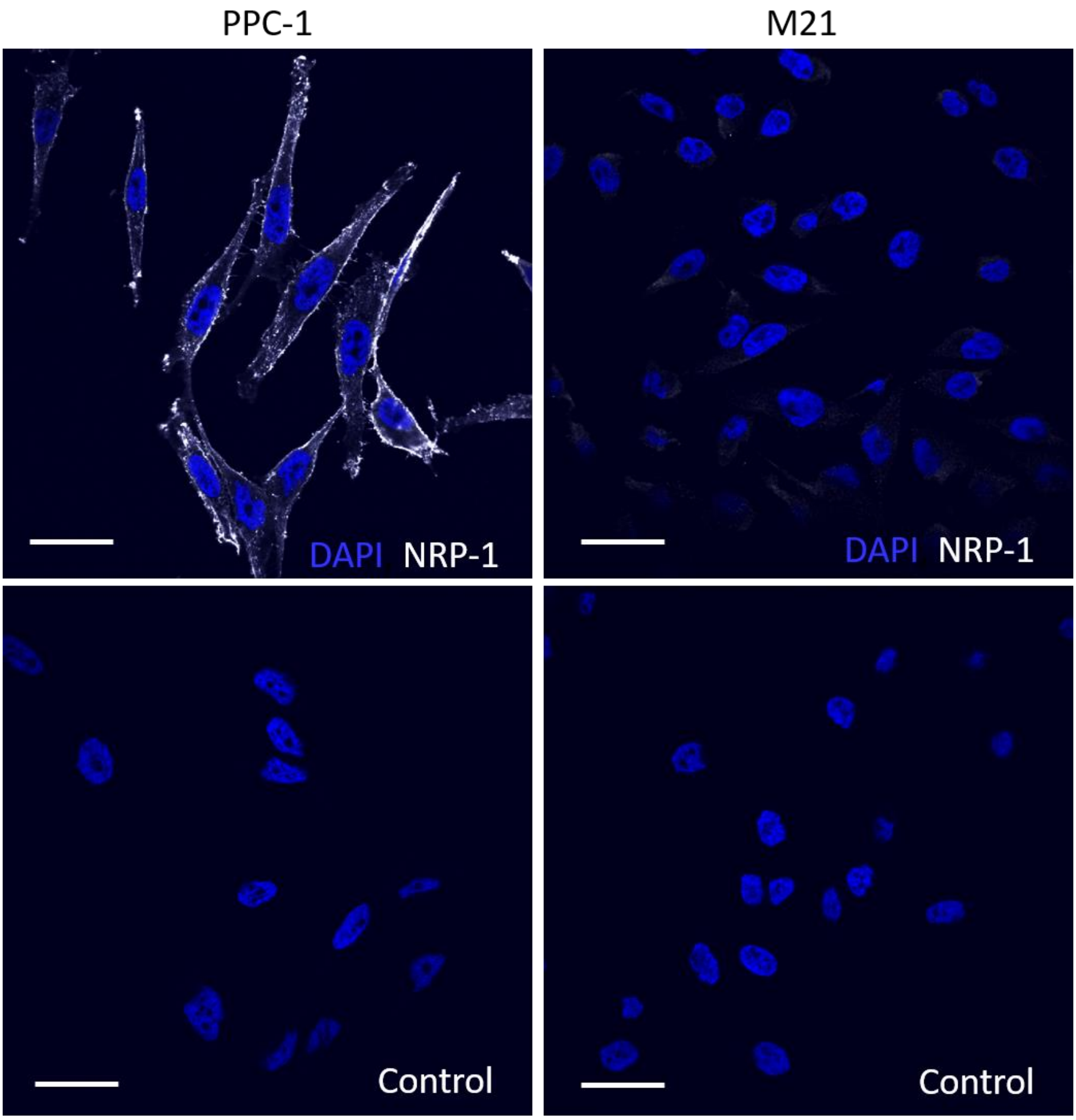

Figure S20. Superficial NRP-1 expression in PPC-1 and M21 cells. Fluorescence confocal microscopy of PPC-1 and M21 cells stained with anti-NRP-1 antibody and DAPI. PPC-1 cells contain high amount of NRP-1 protein whereas M21 cells lack of NRP-1. As a control, cells are stained only with secondary antibody. Scale bar=50 µm.

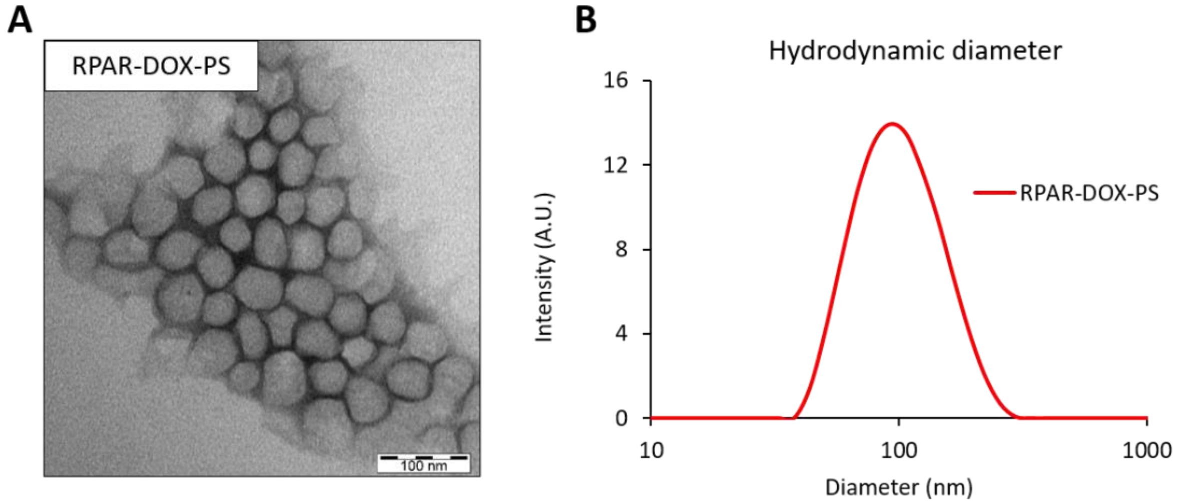

C

Sample	Size (nm) ± SD	PDI	Z-pot (mV)	[DOX] (μM)	EE (%)
RPAR-DOX-PS	113 ± 67	0.179	0.1	57 ± 6	0.6 ± 0.1

Figure S21. Characterization of RPAR-DOX-PS. (A) Image of the RPAR-DOX-PS acquired by TEM. **(B)** Hydrodynamic diameter of RPAR-DOX-PS measured by DLS. **(C)** DOX concentration and encapsulation efficiency (EE%).

A

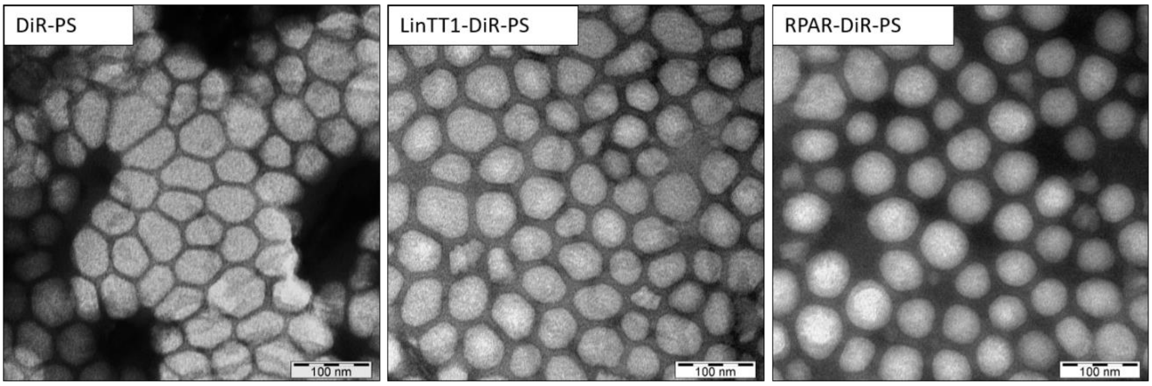

B

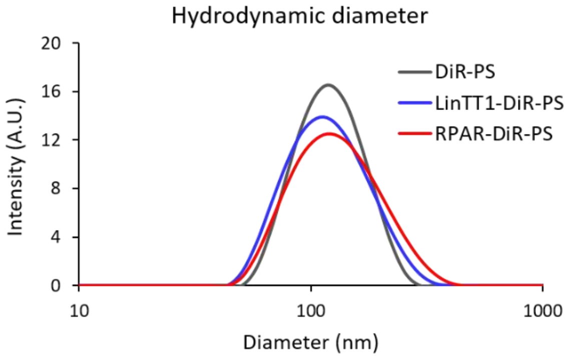

C

Sample	Size (nm) ± SD	PDI	Z-pot (mV)
DiR-PS	114 ± 41	0.091	-1.0
LinTT1-DiR-PS	111 ± 51	0.15	-0.1
RPAR-DiR-PS	124 ± 61	0.218	1.3

Figure S22. Characterization of DiR-labeled PS. (A) Images of DiR-PS acquired by TEM. **(B and C)** Size distribution, PDI, and Zeta potential of DiR-PS samples**.**

.

A

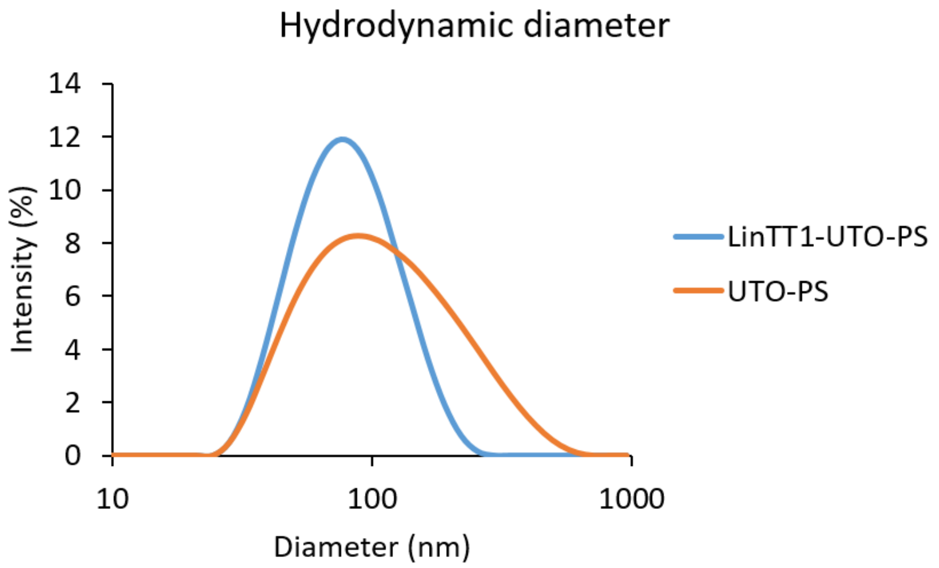

B

Sample	Size (nm) ± SD	PDI	Z-pot (mV)	[UTO] (μM)
LinTT1-UTO-PS	79 ± 110	0.24	-6.5	476
UTO-PS	95 ± 109	0.26	-1.6	506

Figure S23. Characterization of LinTT1-UTO-PS and UTO-PS. (A and **B)** Size distribution, PDI, and zeta potential of UTO-loaded PS samples.

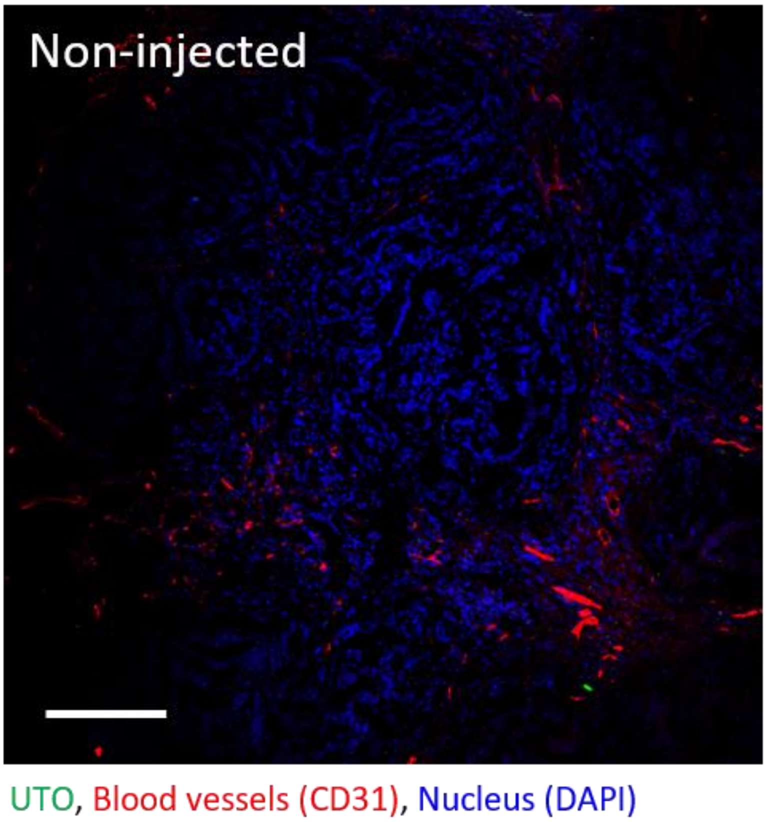

Figure S24. Confocal fluorescence image of sections of MCF10CA1a tumors treated with PBS as a control. Tissues were collected at 24 h post-injection, sectioned, and immunostained for CD31 and stained with DAPI. Green: UTO; red: blood vessels (CD31); blue: nuclei (DAPI). Scale bar=200 µm. Representative image of 3 different tumors.

Table 1. Table of the P-values of viable PPC-1 and M21 cells after 30 min treatment with PS formulations at 2 µM of UTO or DOX and 48 h follow-up incubation. P ≤ 0.0001 - ****; P ≤ 0.001 - ***; P ≤ 0.01 - **; P ≤ 0.05 - *; P > 0.05 – ns.

	RPAR-UTO-PS (PPC-1)	UTO-PS (PPC-1)	PS (PPC-1)	UTO (PPC-1)	DOX (PPC-1)	RPAR-UTO-PS (M21)	UTO-PS (M21)	UTO (M21)	PS (M21)	DOX (M21)
RPAR-UTO (PPC-1)		0.000	0.000	0.000	0.000	0.000	0.000	0.000	0.000	0.000
UTO-PS (PPC-1)	0.000		0.004	0.020	0.102	0.005	0.008	0.173	0.000	0.118
PS (PPC-1)	0.000	0.004		0.540	0.173	0.995	0.820	0.102	0.127	0.151
UTO (PPC-1)	0.000	0.020	0.540		0.444	0.544	0.699	0.294	0.037	0.399
DOX (PPC-1)	0.000	0.102	0.173	0.444		0.175	0.253	0.773	0.006	0.938
RPAR-UTO (M21)	0.000	0.005	0.995	0.544	0.175		0.825	0.103	0.126	0.153
UTO-PS (M21)	0.000	0.008	0.820	0.699	0.253	0.825		0.156	0.082	0.223
UTO (M21)	0.000	0.173	0.102	0.294	0.773	0.103	0.156		0.003	0.833
PS (M21)	0.000	0.000	0.127	0.037	0.006	0.126	0.082	0.003		0.005
DOX (M21)	0.000	0.118	0.151	0.399	0.938	0.153	0.223	0.833	0.005	

References

[1] D. Horton, W. Priebe, M. Sznaidman, *Carbohydr. Res.* **1989**, *187*, 145–148.
[2] A. Uustare, I. Ogibalov, O. Tšubrik, A. Tasa, U. Mäeorg, A. Rinken, *WO2017207540A1*, **2017**.
[3] L. Simón-Gracia, P. D. Scodeller, S. S. S. S. Fuentes, V. G. V. G. Vallejo, X. Ríos, E. S. Sebastián, V. Sidorenko, D. Di Silvio, M. Suck, F. De Lorenzi, L. Y. L. Y. Rizzo, S. Stillfried, K. Kilk, T. Lammers, S. E. S. E. Moya, T. Teesalu, E. San Sebastián, V. Sidorenko, D. Di Silvio, M. Suck, F. De Lorenzi, L. Y. L. Y. Rizzo, S. von Stillfried, K. Kilk, T. Lammers, S. E. S. E. Moya, T. Teesalu, *Oncotarget* **2018**, *9*, 18682–18697.
[4] A. Faustino-Rocha, P. A. Oliveira, J. Pinho-Oliveira, C. Teixeira-Guedes, R. Soares-Maia, R. G. Da Costa, B. Colaço, M. J. Pires, J. Colaço, R. Ferreira, M. Ginja, *Lab Anim. (NY).* **2013**, *42*, 217–224.

Author Contributions

LSG, VS, AU, and IO performed experimental work and data analysis. LSG, VS, and TT wrote the manuscript. LSG and TT advised on experimental design, scope, and editing of the final manuscript. LSG and VS contributed equally to this work. AT and OT advised on data analysis and revision of the manuscript. All authors have given approval to the final version of the manuscript.
